# Convergence-divergence circuits for multimodal integration of innate and learned opponent valences

**DOI:** 10.3389/fnsys.2026.1822122

**Published:** 2026-05-07

**Authors:** Wenjing Wang, Yaokai Yang, Qiong Liu, Yunming Gao, Qiantao Lv, Kaiqi Zhang, Jing Ning, Yi Sun

**Affiliations:** 1 Fudan University, Shanghai, China; 2 Key Laboratory of Growth Regulation and Translational Research of Zhejiang Province, Research Center for Industries of the Future, School of Life Sciences, Westlake University, Hangzhou, China; 3 Westlake Laboratory of Life Sciences and Biomedicine, Hangzhou, China; 4 Institute of Basic Medical Sciences, Westlake Institute for Advanced Study, Hangzhou, China; 5 Zhejiang University, Hangzhou, China

**Keywords:** connectomics, convergence-divergence circuit, dendritic computation, multimodal integration, superior protocerebrum, valence

## Abstract

Valence detection in complex environment is critical for natural behaviors like foraging. Previous studies have explored valence processing in brain regions like lateral horn (LH) and mushroom body (MB) using simple synthetic stimuli in *Drosophila*. However, the neural basis for valence detection of natural objects in complex contexts remains unclear. Here, by brain-wide connectome analysis, we identified the evolutionarily conserved superior protocerebrum (SP) that integrates brain-wide multimodal inputs mainly via LH and MB, and sends widespread outputs particularly to the central complex (CX). This forms a convergence-divergence circuit resembling an autoencoder architecture, with SP as the bottleneck integrating multimodal information into low-dimensional valence signals. Specifically, SP input LH neurons integrate ethologically related innate valences for robust valence detection in natural environments, and the integration can be unimodal, such as that of diverse odors signaling food, or multimodal, such as that of wind and temperature signaling lousy weather. Opponent valences of attraction and aversion are further integrated into SP for complex valence detection. MB learned valences are also integrated into SP to update LH innate valences with recent experience for flexible valence detection. Attractive and aversive valences, either innate or learned, are integrated via excitatory and inhibitory synapses, respectively to form complex valence signals in a single SP neuron. Organized synaptic compartments support dendritic computation, with SP neurons exhibiting opposite synaptic organizations for opponent valences, indicating dendritic integration for complex valence detection. Our study highlights the importance of SP in multimodal opponent valence integration and suggests generalizable network and dendritic structures for complex valence processing.

## 1 Introduction

During natural behaviors like foraging and social interactions in the complex environment, animals must parse contextual cues to extract valence information and integrate it with their internal goals and states to plan appropriate actions (Keleman et al., 2012; Kohl et al., 2013; Sayin et al., 2019; Lin et al., 2022; Münch et al., 2022; Ning et al., 2022; Taisz et al., 2023; Cazalé-Debat et al., 2024; Gautham et al., 2024). These contextual cues are often complex and multisensory. For example, hungry animals during foraging must process food cues, which are multisensory objects characterized by distinct appearances, textures, and scents that convey their identities and valences. Valences of some cues are innate, such as the attractive scent of yeast or the aversive smell of toxic bacteria, whereas others are acquired through experience, such as visual cues linked to unpleasant food. Internal states further modulate the valences of food, for example, food becomes more attractive when the animal is hungry. How animals integrate innate and learned valences in dynamic multimodal external contexts while accounting for internal states to plan actions is remarkable yet elusive.

Processing multisensory contextual cues for valence detection in complex environment requires integrating multimodal information distributed across multiple brain regions. Understanding how different brain regions interact is a fascinating yet challenging question. The recent completion of the whole-brain connectome for the numerically tractable *Drosophila* brain (Zheng et al., 2018; Scheffer et al., 2020; Dorkenwald et al., 2021, 2024; Winding et al., 2023; Eckstein et al., 2024) – complemented by advanced manipulation and measurement tools and a rich behavioral repertoire – offers an ideal model for investigating inter-regional interactions, where dissecting the structural basis is the first step.

*Drosophila* central brain regions lateral horn (LH) and mushroom body (MB) are implicated in innate and learned valence processing, while the central complex (CX) has been implicated in goal-directed action selection informed by valence (Hulse and Jayaraman, 2020; Modi et al., 2020; Wilson, 2023; Fulton et al., 2024), making them candidate inputs and outputs for the interaction question. Theoretical and experimental studies are starting to uncover the interactions between these regions (Dolan et al., 2018; Sun et al., 2020; Matheson et al., 2022; Goulard et al., 2023), and direct inter-regional connectivity between MB, LH, and CX has been examined (Frechter et al., 2019; Li et al., 2020; Hulse et al., 2021; Winding et al., 2023). However, the circuit architecture integrating LH, MB, and CX is unclear. In particular, it remains unknown whether an interneuron network exists to route information between these three regions, and if so, where it is located. Dissecting this interneuron network may elucidate complex valence integration in natural environments.

Across many arthropod species, the evolutionarily conserved superior protocerebrum (SP) sits anatomically among MB, LH, and CX, making it a prime candidate for the interneuron network (Heinze et al., 2012; Phillips-Portillo and Strausfeld, 2012; Ito et al., 2014; El Jundi et al., 2018; von Hadeln et al., 2019; Hensgen et al., 2020; Kaiser et al., 2022). We focused on SP connectivity with these key regions, and found that SP heavily innervates LH, MB, and CX, forming convergence-divergence network, an architecture resembling autoencoders in machine learning (Lecun and Soulie Fogelman, 1987; Kramer, 1991; Goodfellow et al., 2014). We further identified synaptic distribution patterns that underlie dendritic computation in this convergence-divergence network. Our findings reveal that by integrating innate and learned opponent valences from diverse sensory modalities, convergence-divergence circuit design robustly and flexibly transforms high-dimensional sensory inputs into low-dimensional valence signals, which can be broadcasted for adaptive behavior in natural settings. These results are consistent between the hemibrain (Scheffer et al., 2020) and the FAFB/FlyWire (Zheng et al., 2018; Dorkenwald et al., 2024) connectome datasets, indicating the generality.

## 2 Materials and methods

### 2.1 Datasets

We used the morphology, connectivity, and synapse position data from hemibrain (v1.2.1) (Scheffer et al., 2020) and FlyWire (v783) (Schlegel et al., 2024) for this manuscript.

### 2.2 Nomenclature

For the anatomical nomenclature of brain regions in this manuscript, we used the systematic nomenclature of the insect brain by the Insect Brain Name Working Group (Ito et al., 2014). We used the nomenclature for LH, MB, and FB neurons, as well as OPN, VPN, WEDPN, DN, and modulatory neurons based on published studies (Wu et al., 2016; Dolan et al., 2019; Li et al., 2020; Hulse et al., 2021; Schlegel et al., 2021).

### 2.3 Threshold setting

In the hemibrain and FlyWire datasets, the number of synapses between two neurons refers to the number of postsynaptic sites on the postsynaptic neuron (Scheffer et al., 2020; Dorkenwald et al., 2024). To eliminate spurious connections, we filtered out connections with less than two synapses between two neurons in all connectivity analyses.

### 2.4 Definition of SP intrinsic neuron (SPIN)

#### 2.4.1 Superior protocerebrum (SP)

**FIGURE 1:**
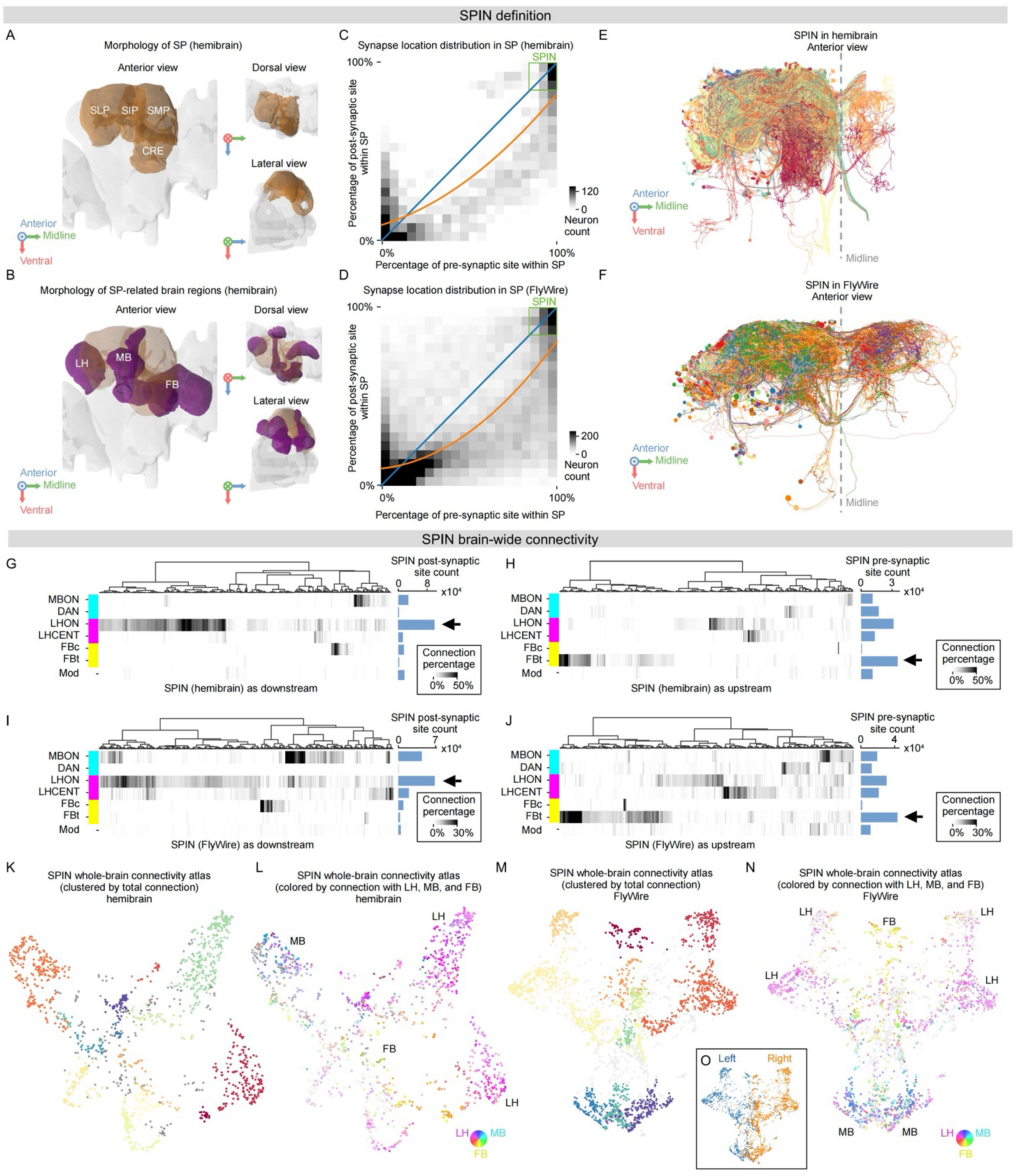
Definition and connectivity of SP intrinsic neurons (SPINs). **(A)** Superior protocerebrum (SP) neuropiles in the right hemisphere of *Drosophila* brain (based on hemibrain template), including superior lateral protocerebrum (SLP), superior intermediate protocerebrum (SIP), superior medial protocerebrum (SMP), and crepine (CRE), highlighted in brown and shown in anterior, dorsal, and lateral views. See Supplementary Table 1 for abbreviations. **(B)** SP-related neuropiles, including the mushroom body (MB), lateral horn (LH), and fan-shaped body (FB) in the fly brain (based on hemibrain template), highlighted in magenta and shown in anterior, dorsal, and lateral views. **(C,D)** SP-related neuron synapse location distribution in SP with binning (5% bin size) in hemibrain **(C)** and FlyWire **(D)**. The horizontal axis shows the percentage of pre-synaptic sites inside SP, while the vertical axis shows the percentage of post-synaptic sites inside SP. Blue curve, y = x; orange curve, polynomial fitting curve of SP-related neuron post-synaptic site versus pre-synaptic site distributions in SP, hemibrain **(C)**, y = 0.31x^2^ + 0.45x + 0.08, R^2^ = 0.67; FlyWire **(D)**, y = 0.62x^2^ + 0.10x + 0.10, R^2^ = 0.63. The green squares denote SPINs. Also see Supplementary Figure 1H for synapse location distribution of all SP-related neurons without binning. **(E,F)** Anterior view morphology of 500 randomly selected SPINs in the right hemisphere in hemibrain **(E)** and FlyWire **(F)**, randomly colored for each SPIN. **(G–J)** SPIN connectivity with major cell groups in MB, LH, FB, and modulatory neurons as upstream in hemibrain **(G)** or FlyWire **(I)** or downstream in hemibrain **(H)** or FlyWire **(J)**, shown as the percentage of synaptic contribution for each SPIN. SPINs are clustered by connectivity. The summated synaptic counts of each major cell group with SPINs are displayed on the right of each panel (blue bars). LHON and FBt (black arrows) are the dominant upstream and downstream cell groups of SPINs. **(K,L)** SPIN whole-brain connectivity atlas (hemibrain) via UMAP. Each dot denotes a single SPIN. SPINs are color-coded by 15 clusters based on brain-wide connectivity **(K)** and connectivity preference to LH, MB, or FB **(L)**. **(M,N)** SPIN whole-brain connectivity atlas (FlyWire) via UMAP. Each dot denotes a single SPIN. SPINs are color-coded by 12 clusters based on brain-wide connectivity **(M)** and connectivity preference to LH, MB, or FB **(N)**. **(O)** Hemisphere distribution on SPIN whole-brain connectivity atlas (FlyWire). SPINs in the left or right hemisphere are colored blue and orange, respectively.

Crepine (CRE), which surrounds the MB medial lobe, is traditionally not considered part of superior neuropils. We found a strong connection between CRE and superior neuropils (Supplementary Figure 1B). CRE and SIP warp around MB horizontal and vertical lobes, respectively (Figure 1B). We reasoned that such an organization, which is evolutionarily conserved (El Jundi et al., 2010, 2018; Phillips-Portillo and Strausfeld, 2012; Ito et al., 2014; Habenstein et al., 2023), enhances wiring efficiency with MB. Indeed, CRE is connected with MB as heavily as superior neuropils (Supplementary Figure 1C; Aso et al., 2014a). Similarly, both CRE and SMP are adjacent regions and strong connection partners of the fan-shaped body (FB) (Supplementary Figure 1C). Reasoning that having intrinsic neurons is a signature of an independent region, we looked for intrinsic neurons in CRE. We defined CRE-related neurons as “traced” fragments with at least two pre- or post-synaptic sites located in CRE in the hemibrain dataset. We calculated the pre- and post-synaptic site counts of all CRE-related neurons in each brain region, and plotted their synapse distribution in the brain regions connected with more than half of the CRE-related neurons in Supplementary Figure 1B. We found no intrinsic neurons in CRE, and most CRE-related neurons are CRE-SMP or CRE-SMP-SIP neurons (other than CRE innervating MBON), indicating that CRE is anatomically associated with superior neuropils. Finally, we quantified the connectivity strengths between major neuron types in CRE, SP, and MB/FB (Supplementary Figure 1C), and found that CRE and SMP are both crucial hubs in this network, further indicating that CRE shares a similar pattern of connectivity with superior neuropils.

#### 2.4.2 SP intrinsic neuron (SPIN) in hemibrain

To decompose the compartments in SP, we first collected all fragments in the right hemisphere of the hemibrain dataset that transmit information through SP (including SMP, SIP, SLP, and CRE) with at least two pre- or post-synaptic sites. There are 11288 fragments with pre-/post- synaptic sites in unilateral SP (right side), in which 9384 fragments are “well-traced” (Supplementary Figure 1D), since the left hemisphere in hemibrain is incomplete. To distinguish well-reconstructed neurons from all fragments, we then quantified the relation between total synapse counts of each continuous fragment and the number of fragments in this dataset, and found that both populations consist of two regimes. The left regime shows a weaker relation between synapse counts and fragments count, representing the partially-traced short fragments (Supplementary Figure 1D); while the right regime exhibits a stronger relation. Thus, we set the approximate turning point (75 synaptic sites, green dashed-line in Supplementary Figure 1D) as a threshold between properly traced neurons and partially-traced short fragments. We defined the properly-traced neurons as SP-related neurons. By calculating the distribution of SP-related neurons synapse percentage in SP (Figure 1C, Supplementary Figure 1H), we found that a subset of SP-related neurons exhibited a strong preference (over 85% of synapses) for the SP region, as highlighted by the green rectangle in Figure 1C. We defined this SP-related neuron population as the SP intrinsic neurons (SPINs). After analyzing the statistical distribution of the pre- and post-synaptic sites of all SPINs (Supplementary Figure 1E, upper panel), we found that the threshold of 75 was very close to the peak of the distribution for pre-synaptic sites, and seemed to truncate the histogram. Thus, we added those neurons that belong to types with at least one neuron counted as SPINs but with pre-synaptic sites less than 75 to SPINs, making the histogram more natural (Supplementary Figure 1E, lower panel). To avoid confusion, we manually examined all SPINs, and removed neurons belonging to reported cell types, such as LHONs, descending neurons (DNp25), and known modulatory neurons like PPL107. The final count for the total number of neurons in the SPIN population in the right hemisphere of hemibrain is 1411.

#### 2.4.3 SP intrinsic neuron (SPIN) in FlyWire

We also decomposed the SP neurons in FlyWire. Since fragments in FlyWire lack tracing result estimation as in hemibrain, we used all fragments across the brain to derive the whole-brain synapse count distribution, and to distinguish well-traced neurons in FlyWire. We reasoned that the whole-brain synapse count distribution in FlyWire comprises two populations, including partially-traced fragments and well-traced neurons. We found two peaks in both pre-synaptic and post-synaptic site count distribution curves of FlyWire, corresponding to the synapse distribution of partially-traced fragments and well-traced neurons, respectively (Supplementary Figure 1F). Thus, we set the intersection point (40 synapses, see dashes in Supplementary Figure 1F) between the two peaks in both pre- and post-synaptic site count distribution, as the threshold to filter out partially-traced fragments in FlyWire. We only considered the fragments containing more than 40 pre- and post-synaptic sites for the following analyses in FlyWire. Next, we defined SP-related neurons by collecting all fragments that transmit information through SP (including SMP, SIP, SLP, and CRE) with at least two pre- or post-synaptic sites in FlyWire. We calculated the pre-/post-synaptic site percentage in SP of each SP-related neuron in FlyWire, and found a similar distribution as in hemibrain (Figures 1C,D, Supplementary Figure 1H). We also found a subset of SP-related neurons in FlyWire exhibiting a strong preference (over 85% of synapses) for the SP region by adding the synapse percentage of the left and right SP together. We defined this SP-related neuron population as the SPIN in FlyWire, as highlighted by the green rectangle in Figure 1D. The final SPIN count in FlyWire is 2844 neurons in the left and right hemispheres. This number is very close to the double of 1411 for the hemibrain dataset. There are 1479 and 1365 SPINs in the right and left hemispheres, respectively (Figure 1O).

### 2.5 Connectivity

#### 2.5.1 Clustering methods

We mainly performed hierarchical clustering on SPINs connectivity analyses based on their normalized connectivity strength matrix with neurons or regions. We used the logarithmic function for SPIN intrinsic connectivity matrix normalization and used the connectivity percentage of each SPIN to represent the connectivity strength with other neuron types or regions. We first used the Bray-Curtis distance (Bray and Curtis, 1957) during clustering to calculate the pairwise distances between SPINs. The distance *d*(*u*,*v*) between two SPINs *u* and *v* is:


$$d\,\left(u,v\right)=\frac{\sum_{i}\left|u_{i}-v_{i}\right|}{\sum_{i}\left|u_{i}+v_{i}\right|}$$

where *u* and *v* are vectors of brain-wide connectivity of a single SPIN. Then, we used the Ward variance minimization algorithm (Ward, 1963) to compute the distance between clusters in hierarchical clustering, due to its superior performance on large datasets. The algorithm begins with a forest of clusters yet to be used in the hierarchy being formed. When two clusters *s* and *t* from this forest are combined into a single cluster *u*, *s* and *t* are removed from the forest, and *u* is added to the forest. The algorithm stops when only one cluster is left in the forest, and this cluster becomes the root. We set *s* and *t* to be combined to form cluster *u*, and *v* is an unused cluster in the forest. The distance *d*(*u*,*v*) between two clusters *u* and *v* is computed as follows:


$$d\,\left(u,v\right)=\sqrt{\frac{\left|v\right|+\left|s\right|}{T}\,d\,{\left(v,s\right)}^{2}+\frac{\left|v\right|+\left|t\right|}{T}\,d\,{\left(v,t\right)}^{2}-\frac{\left|v\right|}{T}\,d\,{\left(s,t\right)}^{2}}$$

where *T*=|*u*| + |*s*| + |*t*|, and |*| refers to the cardinality of its argument. The *d*(*i*,*j*) entry corresponds to the distance between cluster *i* and *j* in the original forest.

#### 2.5.2 SPIN extrinsic connectivity with brain regions

We first studied SPIN input and output regions (Supplementary Figures 1I,J). Since SPIN synapses are concentrated in the SP, we traced the SPIN first stage upstream and downstream locations in the brain. We calculated the distribution of SPIN upstream dendrite and downstream axon counts by brain regions in hemibrain (Supplementary Figure 1I) and FlyWire (Supplementary Figure 1J), respectively, and sorted the brain regions in descending order. These panels showed that SPINs have a special preference for LH, MB, and FB, as highlighted in purple in Supplementary Figures 1I,J.

#### 2.5.3 Major cell groups

From the above analysis, we found that the main upstream and downstream regions of SPINs are MB, FB, and LH, whose major cell groups were already known (Li et al., 2020; Hulse et al., 2021; Schlegel et al., 2021). Therefore, we further investigated the relationship between SPINs and these brain regions through major cell groups. In the MB, we considered the main output cell group MBON, and the modulatory DAN. Similarly, we selected LHON and LHCENT for LH, and selected FBt and FBc in FB, such as FC, FS, PFR, PFG, and PFL. Intrinsic neurons, such as Delta neurons in FB, were not considered. As to the sensorimotor system, we examined the olfactory projection neuron (OPN) (Schlegel et al., 2021), visual projection neuron (mainly LC and LT neurons) (Wu et al., 2016; Schlegel et al., 2024), WED projection neuron (WEDPN) (Schlegel et al., 2021), and descending neurons (DN) (Namiki et al., 2018). In addition, large modulatory neurons (e.g., PPL107, 5-HTPMPD01, OA-VPM3, AstA1) (Deng et al., 2019) and some reported neurons passing through SP, such as pC1 (Hoopfer et al., 2015) and oviIN (Wang F. et al., 2020), were also examined.

#### 2.5.4 SPIN extrinsic connectivity with major cell groups

We calculated the connectivity percentage of SPINs with major cell groups in hemibrain and FlyWire, and selected SPINs with strong connectivity preferences for these cell groups by setting a threshold of their connectivity strength. We defined SPINs with over 5% pre- or post-synaptic sites with any major cell groups as having preference with this cell group. We collected this SPIN subset and clustered them based on their connectivity percentage with all major cell groups by hierarchical clustering (Figures 1G–J, Supplementary Figures 6A,B).

#### 2.5.5 SPINs clusters with regional preferences

We defined SPINs with regional preferences as SPINs having strong connectivity (>5% of this SPIN total input or output) with either major cell group in that region. We then performed hierarchical clustering on SPINs with regional preference (e.g., LH-related SPINs) based on their connectivity percentage with major cell groups in that region, and defined SPIN clusters by cutting the hierarchical tree dynamically.

### 2.6 UMAP

#### 2.6.1 UMAP generation

We used the uniform manifold approximation and projection (UMAP) algorithm (McInnes and Healy, 2018) to reduce the dimensionality of the SPIN connectivity matrix to visualize these populations. UMAP is a k-neighbor-based graph learning technique for dimensionality reduction that favors the preservation of local distances over global distances (McInnes and Healy, 2018). UMAP is widely used (Becht et al., 2019; Cao et al., 2019) for its better visualization quality that preserves more of the global structure and its superior run-time performance than t-SNE (McInnes and Healy, 2018). The first step of the UMAP algorithm is to generate a weighted k-neighbor graph by Laplacian eigenmaps that approximate the uniform manifold of the dataset. Next, we used fuzzy simplicial sets to transform the graph from metric space to fuzzy topological representations and computed a low-dimensional layout of this graph. This layout was optimized by stochastic gradient descent. To run UMAP on the SPIN connectivity, we first generated the SPIN upstream and downstream connectivity matrices, by counting the synapse counts of SPIN and its post- and pre-synaptic neurons for the upstream matrix and downstream matrix, respectively. We then normalized the upstream or downstream connectivity vector in the upstream or downstream connectivity matrix of each SPIN with the total number of post-/pre-synaptic sites of this SPIN. We concatenated the upstream and downstream connectivity vectors of each SPIN into a single line vector as the combined connectivity vector of each SPIN. We generated a combined connectivity matrix of all SPINs using these line vectors. We performed UMAP on the combined connectivity matrix. To generate the weighted graph of SPINs, we identified a proper representation of SPIN distances with each other. Here, we used the Bray-Curtis distance (Bray and Curtis, 1957) to calculate the distance between each of the two SPINs and generated the distance matrix of SPINs. Next, we tested different parameter combinations (e.g., neighbor numbers, layout dimensions) of UMAP to better approximate the real manifold of the SPIN graph. After thousands of rounds of testing, we found the best combination of parameters for UMAP rendering in hemibrain is, neighbor = 36, layout dimension = 3, mindist = 0.1, with a random initialization factor of layout graph = 50. As to SPINs in FlyWire, we used the same method (Bray-Curtis distance) to calculate the distance matrix of SPIN, and the best combination of UMAP parameters in FlyWire is, neighbor = 32, layout dimension = 3, mindist = 0.13, with the random initialization factor of layout graph also equals 50. To assess the robustness of the UMAP embedding, we systematically varied n_neighbors ([24, 32, 40, 48]) and min_dist ([0.1, 0.15, 0.2, 0.25]) parameters and confirmed that the backbone structure is stable across parameter combinations in hemibrain (Supplementary Figure 2A) and FlyWire (Supplementary Figure 2E). We mainly used the 2D projection in the Component 1/2 plane of 3D UMAP to show the SPIN distribution in UMAP space (e.g., Figures 1K–O). We also used 3D view UMAP for videos. Each node in the UMAP atlas denotes a SPIN neuron.

#### 2.6.2 Clustering on UMAP

We performed clustering on UMAP with HDBSCAN (McInnes et al., 2017), a density-based algorithm particularly suitable for 3D dot array data after UMAP dimensionality reduction, rather than k-means and other traditional clustering ways. In hemibrain, we clustered SPINs into 15 clusters based on this result and colored the points according to the clusters (Figure 1K), while we clustered SPINs into 12 clusters in FlyWire (Figure 1M). It is worth noting that one feature of HDBSCAN is that it can refuse to cluster some points and classify them as noise (McInnes et al., 2017). We colored the noise points as gray.

To assess whether SPIN clusters represent discrete types or a continuous gradient, we computed the local density of each SPIN in the original high-dimensional connectivity space. For each SPIN, we calculated the mean Bray-Curtis distance to its k nearest neighbors (*k* = 15, matching the min_samples parameter used in HDBSCAN) in the concatenated upstream–downstream connectivity matrix (the same matrix used for UMAP embedding). Local density was defined as the inverse of the mean k-NN distance and rendered on UMAP (Supplementary Figure 2B for hemibrain, Supplementary Figure 2F for FlyWire). To visualize the density landscape, we additionally computed a 2D kernel density estimate (KDE, Scott’s bandwidth) on the UMAP coordinates (Supplementary Figure 2C for hemibrain, Supplementary Figure 2G for FlyWire).

#### 2.6.3 Rendering on UMAP

**FIGURE 2:**
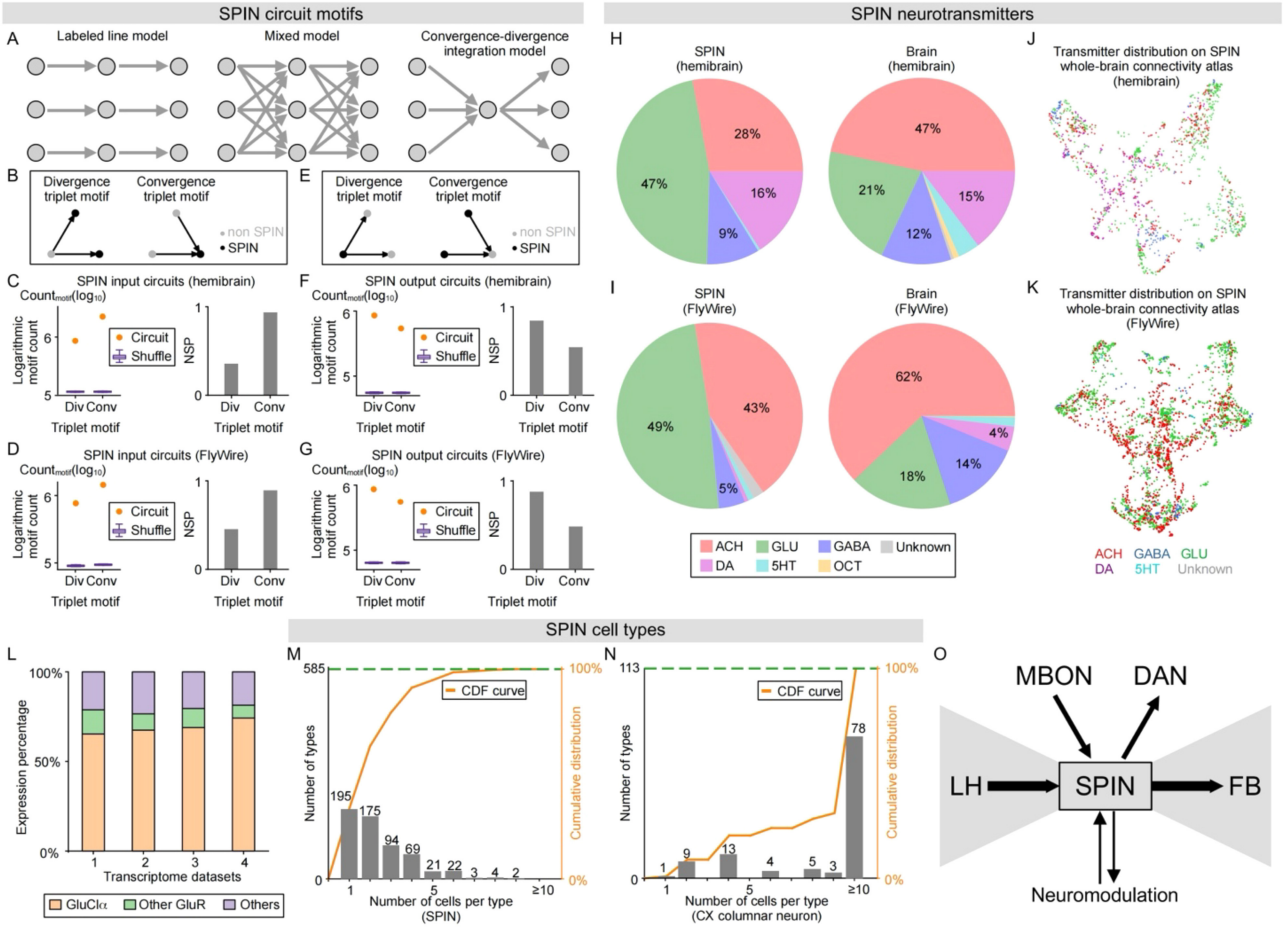
Circuit motif, neurotransmitter, and cell type analysis of SPINs. **(A)** Hypothesized SPIN circuit models, including labeled-line model (left), mixed model (middle), and convergence-divergence integration model (right). **(B)** Divergence and convergence triplet motifs in SPIN input circuits. **(C,D)** Triplet motif analysis of SPIN input circuits in hemibrain **(C)** and FlyWire **(D)**. The left and right panels show the counts and network significance profiles (NSP) of triplet motifs in SPIN input circuits. Orange dots in the left panels denote the counts of each triplet motif in SPIN input circuits, while the blue box plots show the count distribution of 100 times of random reshuffling (boxes look like bars, same as follows). See Methods for details of the analysis. **(E)** Divergence and convergence triplet motifs in SPIN output circuits. **(F,G)** Triplet motif analysis of SPIN output circuits in hemibrain **(F)** and FlyWire **(G)**. Left and right panels show the counts and network significance profiles (NSP) of triplet motifs in SPIN output circuits. Orange dots in the left panels denote the counts of each triplet motif in SPIN output circuits, while the blue box plots show the count distribution of 100 times of random reshuffling. **(H,I)** Neurotransmitter distribution of SPINs (left) and whole brain neurons (right) in hemibrain **(H)** and FlyWire **(I)**. **(J,K)** SPIN neurotransmitter distribution on whole-brain connectivity atlas in hemibrain **(J)** and FlyWire **(K)**, color-coded by neurotransmitters. Also see distributions of each transmitter in Supplementary Figure 5. **(L)** Glutamate receptor subtypes in *Drosophila* central brain, based on single-cell sequencing datasets [1 from Davie et al. (2018); 2 from Janssens et al. (2022); 3 from Croset et al. (2018); 4 from Li et al. (2022)]. Most of the glutamate receptors are the inhibitory GluClα. **(M,N)** Histogram and cumulative distribution (orange curve) of the numbers of cells in each cell type of all SPIN **(M)** and central complex **(N)** cell types in hemibrain. The numbers of cells in each type are labeled on top of each bar. **(O)** Working model of SPIN circuit architecture mediating MB-modulated LH to FB network.

**FIGURE 3:**
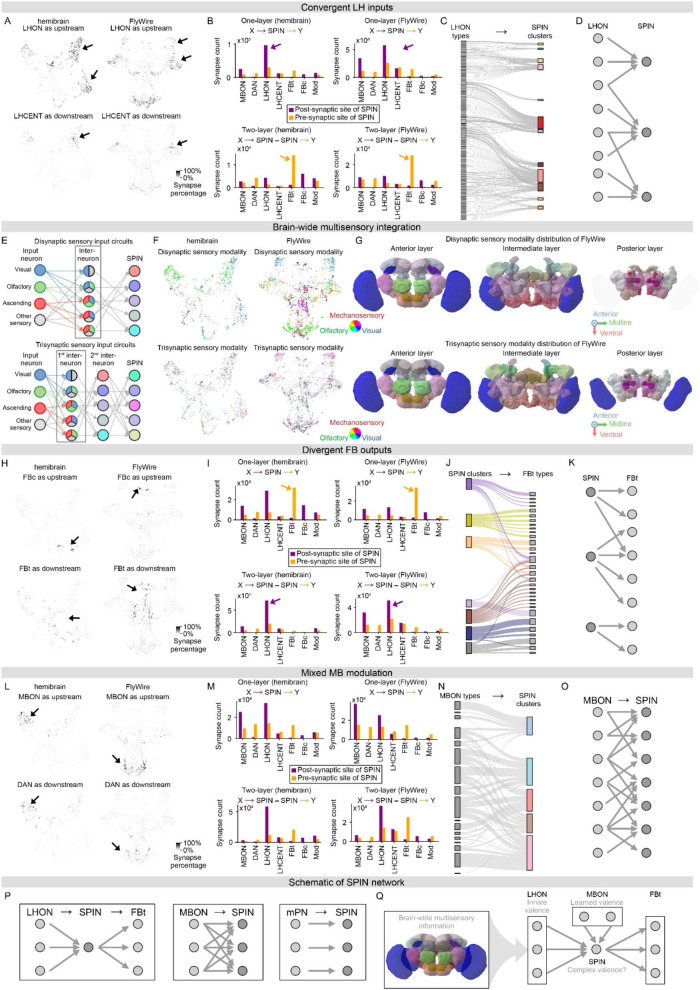
SP intrinsic neurons (SPIN) input and output circuits. **(A)** SPIN connectivity strengths with LHON as upstream (upper) or LHCENT as downstream (lower) in hemibrain (left) and FlyWire (right), shown on SPIN whole-brain connectivity atlas. Each dot denotes a single SPIN. The contrast of the dots denotes the SPIN connectivity strength (calculated by the percentage of synaptic site, see Methods). Arrows highlight major LH-related SPIN populations. **(B)** One- (upper row, X→SPIN→Y) and two- (lower row, X→SPIN→SPIN→Y) layer connectivity preference of LH-related SPINs with major cell groups as upstream (magenta) or downstream (orange), based on synapse counts in hemibrain (left column) and FlyWire (right column). Magenta and orange arrows highlight the leading input and output cell groups, respectively. **(C)** Many-to-one connectivity from LHONs to LH-related SPINs in hemibrain. SPINs are clustered and color-coded by connectivity (see Methods). Connections exceeding 50 synapses are shown. **(D)** Schematic of the convergent (fan-in) architecture of LHON-SPIN circuits. Multiple LHONs converge onto a single SPIN, which then projects to FBt neurons directly or indirectly via intermediate SPINs. **(E)** Schematic of disynaptic (upper) and trisynaptic (lower) sensory input network of SPIN. **(F)** SPIN sensory modality preferences on whole-brain connectivity atlas in hemibrain (left column) and FlyWire (right column). Each dot denotes a single SPIN. SPINs are color-coded by their disynaptic (upper) and trisynaptic (lower) sensory modality input preferences. **(G)** Brain-wide sensory modality preferences of disynaptic (upper) and trisynaptic (lower) multimodal SPIN input circuits in FlyWire. Brain regions are colored by their sensory modality preferences, calculated by the pre-synaptic site percentage from visual, olfactory, and ascending neurons monosynaptically (see Methods). **(H)** SPIN connectivity strengths with FBc as upstream (upper) or FBt as downstream (lower) in hemibrain (left) and FlyWire (right), shown on SPIN whole-brain connectivity atlas. Each dot denotes a single SPIN. Dot contrast denotes SPIN connectivity strength (as the percentage of synaptic sites, see Methods). Arrows highlight major FB-related SPIN populations. **(I)** One- (upper) and two- (lower) layer connectivity preference of FB-related SPINs with major cell groups as upstream (magenta) or downstream (orange), based on synapse counts in hemibrain (left) and FlyWire (right). Magenta and orange arrows highlight the leading input and output cell groups, respectively. **(J)** One-to-many connectivity from FB-related SPINs to FBt neurons in hemibrain. SPINs are clustered and color-coded by connectivity (see Methods). Connections exceeding 50 synapses are shown. **(K)** Schematic of the divergent (fan-out) architecture of SPIN-FBt circuits. A single SPIN projects to multiple FBt neurons across different FB layers, broadcasting convergent LHON information to diverse downstream targets directly or indirectly via intermediate SPINs. **(L)** SPIN connectivity strengths with MBON as upstream (upper) or DAN as downstream (lower) in hemibrain (left) and FlyWire (right), shown on SPIN whole-brain connectivity atlas. Each dot denotes a single SPIN. Dot contrast denotes SPIN connectivity strength (as the percentage of synaptic sites, see Methods). Arrows highlight major MB-related SPIN populations. **(M)** One- (upper) and two- (lower) layer connectivity preference of MB-related SPINs with major cell groups as upstream (magenta) or downstream (orange), based on synapse counts in hemibrain (left) and FlyWire (right). **(N)** Many-to-many connectivity from MBONs to MB-related SPINs in hemibrain. SPINs are clustered and color-coded by connectivity (see Methods). Connections exceeding 50 synapses are shown. **(O)** Schematic of the many-to-many architecture of MBON-SPIN circuits. Multiple MBON types project to multiple SPIN clusters. **(P)** Summary of SPIN circuit motifs, including labeled-line mPN-SPIN circuit, mixed MBON-SPIN circuit, and convergence-divergence integration LHON-SPIN-FBt circuit. **(Q)** LHON/MBON-SPIN-FBt convergence-divergence multimodal integration circuit.

We rendered the UMAP in various ways, including SPIN connectivity preference to LH, MB, and FB (Figures 1L,N), morphological clustering results (Supplementary Figures 5D,E), predicted neurotransmitters (Figures 2J,K, Supplementary Figures 5A,B), connectivity strength to specific neuron groups (e.g., Figure 3A), and sensory modality preferences (Figure 3G) in both hemibrain and FlyWire. We also rendered the UMAP by SPINs location by brain side (Figure 1O) in FlyWire. To visualize the SPIN connectivity preference of LH, MB, and FB (Figures 1L,N), for each SPIN, we first summarized the input and output connectivity percentage with all major cell groups in a brain region (LH, MB, or FB), respectively. Then we choose the larger one of summarized input and output connectivity percentage with this brain region as the SPIN connectivity preference parameter to this brain region. By this, we got a 3-elements vector to represent a SPIN connectivity preference with LH, MB, and FB. We then transformed this vector to a color vector by CMY color code (cyan channel, MB preference parameter; magenta channel, LH preference parameter; yellow channel, FB preference parameter) to visualize the connectivity preference vectors with the three brain regions for each SPIN. For the connection strength rendering (Figures 3A,H,L), we used the connectivity percentage with specific major cell groups to visualize the connectivity strengths. Neurons with higher connectivity percentages are labeled with darker colors. To visualize the SPIN connectivity preference with its disynaptic and trisynaptic sensory inputs in FlyWire (Figure 3F), we first calculated the disynaptic and trisynaptic relative connectivity strength from olfactory, mechanosensory, and visual neurons to SPINs, respectively. After this, we got a 3-elements vector to represent connectivity strength with olfactory, mechanosensory, and visual neurons for each SPIN. We then transformed this vector to a color vector by RGB color code (red channel, mechanosensory connectivity strength; green channel, olfactory connectivity strength; blue channel, visual connectivity strength) to visualize the sensory input preference with each SPIN.

### 2.7 Motif

#### 2.7.1 Shannon entropy analysis

To quantify the diversity of SPIN input and output connectivity as an independent test of the three hypothesized circuit models, we computed the Shannon entropy (Shannon, 1948) of the synaptic weight distribution for each SPIN. For each SPIN, we constructed a weight vector across all its upstream sources (for input circuits) or downstream targets (for output circuits), normalized by total synaptic weight to obtain a proportion vector *p*, and calculated


$$H=-\sum p_{i}\times \log_{2}p_{i},$$

where *p_i_* is the proportion of synaptic weight from source (or to target) *i*. Shannon entropy is bounded between 0 (all weight concentrated on a single partner) and *log*_2_⁡*N* (weight uniformly distributed across *N* partners), and is sensitive to both the number of partners and the evenness of their contributions (Betzel et al., 2024; Moon et al., 2026). To test whether the observed entropy distributions are consistent with specific circuit models, we constructed three null models and applied statistical tests to reject each.

In the labeled-line null, all synaptic weight of each SPIN was assigned to a single randomly chosen partner, simulating a network in which each SPIN is dominated by one source or target (expected *H*  0). To reject this null, we performed a one-sample *t*-test of observed entropy values against 0.

In the mixed-model null, weight was distributed uniformly across all partners of each SPIN, simulating a mixed (all-to-all) network (*H*_*expect*_ = *log*_2_*N*). To reject this null, we computed the entropy deficit (*H*_*observed*_-*log*_2_*N*) for each SPIN and performed a one-sample *t*-test against 0.

In the shuffled null, source or target labels were randomly reassigned across all connections while preserving the degree sequence (number of partners and weight distribution per SPIN), testing whether the observed entropy structure reflects specific wiring rather than a trivial consequence of the number of connections. The shuffled null was repeated 100 times to generate a distribution of mean entropy values. To reject this null, we computed


$$Z\,s\,c\,o\,r\,e=\frac{H_{o\,b\,s\,e\,r\,v\,e\,d}-\mu_{s\,h\,u\,f\,f\,l\,e\,d}}{\sigma_{s\,h\,u\,f\,f\,l\,e\,d}},$$

where μ_*shuffled*_ and σ_*shuffled*_ are the mean and standard deviation of the shuffled null distribution, and derived a two-sided *p*-value from the standard normal distribution.

#### 2.7.2 Circuit motif analysis

We counted the number of triplet motifs in SPIN upstream, downstream circuits, respectively. We first generated possible triplet and motif sets based on the full permutation of directed edges between 3 nodes, and equivalent motifs were then merged into one. Next, we divided the SPIN connectivity matrix into 2 parts: SPIN upstream connectivity matrix (SPIN and its non-SPIN upstream) and SPIN downstream connectivity matrix (SPIN and its non-SPIN downstream). We then simplified the three SPIN connectivity matrices by setting a threshold of connection strength: connections with at least 5 synapses in hemibrain and 2 synapses in FlyWire were included in the triplet motif analysis. The 5 synapse threshold is the most commonly used standard in the *Drosophila* connectomics field for filtering false-positive connections (Li et al., 2020; Hulse et al., 2021; Schlegel et al., 2024). The lower ≥ 2 threshold for FlyWire reflects the ∼5-fold difference in z-resolution between hemibrain (FIB-SEM, ∼8 nm) and FlyWire (ssTEM, ∼40 nm). Because a typical *Drosophila* central brain synapse spans approximately 200–500 nm along the *z*-axis (Scheffer et al., 2020; Buhmann et al., 2021), each synapse is captured across 25–60 serial sections in hemibrain but only 5–12 sections in FlyWire. This reduced sampling causes each biological synapse to be represented by fewer postsynaptic density annotations in FlyWire, resulting in systematically lower synapse counts per connection. To verify that this threshold difference does not affect our conclusions, we repeated the motif analysis on FlyWire at *T* = 5, and the results are consistent with the *T* = 2 analysis (Supplementary Figure 4).

Then we counted the number of each triplet motif in SPIN circuits and calculated the network significance profile (NSP) (Matelsky et al., 2021) of each motif (Figures 2C,D,F,G, right panel to detect motifs significance in SPIN circuits). The network motifs are the subgraph for which the probability of appearing in a real network is greater than in the randomized network. Thus, the null hypothesis is that the number of subgraphs appears not to be significantly different between the real network and the randomized network, while the alternative hypothesis is that the number of subgraphs is significantly different between the real network and the randomized network. So, for each subgraph *i*, the statistical significance is described by the *Zscore*:


$$Z\,s\,c\,o\,r\,e=\frac{N_{r\,e\,a\,l}-N_{r\,a\,n\,d}}{s\,t\,d\,\left(N_{r\,a\,n\,d}\right)},$$

where the *N*_*real*_ is the count of the subgraph *i* appearing in the real network, *N*_*rand*_ and *std*(*N*_*rand*_) are the mean and standard deviation of the subgraph *i* appearance in the randomized network ensemble. In this paper, we generated a random network by reshuffling the real network. The mean and standard deviation of each subgraph appearance were calculated from the results of 100 times reshuffling. The network significance profile (NSP) is defined as a normalized vector of *Zscore*:


$$N\,S\,P_{i}=\frac{Z\,s\,c\,o\,r\,e_{i}}{\sqrt{\sum Z\,s\,c\,o\,r\,e_{i}^{2}}}.$$

### 2.8 Relative connectivity for multi-layer networks

We calculated the relative connectivity matrix of the SPIN multimodal input circuit (e.g., Figure 3F; Li et al., 2020). The relative connectivity strength between SPINs and sensory neurons (e.g., OPNs) is computed by normalizing the upstream connectivity matrix (sensory neurons → intermediate neurons) and downstream connectivity matrix (intermediate neurons → SPINs) by rows and columns respectively, and multiplying these matrices. For each SPIN *i*, its connectivity with sensory neurons (like OPNs) can be described as an upstream matrix U (for OPN i, the upstream vector is *U_i_*) and downstream vector *D_i_*,


$$U_{i}=\left(u_{i\,1},u_{i\,2},\cdots ,u_{i\,j},\cdots ,u_{i\,k}\right)$$

$$D_{i}=\left(d_{i\,1},d_{i\,2},\cdots ,d_{i\,j},\cdots ,d_{i\,k}\right),$$

in which K denotes to the total intermediate neuron number, *u*_*ij*_ denotes the synapse percentage that OPN *i* projected to intermediate neuron *j*, *d*_*ij*_ denotes to the synapse percentage that intermediate neuron *j* projected to SPIN *i*, and *m* denotes to the number of OPNs. Thus, the relative connectivity matrix *C* between all SPINs and OPNs in the brain can be described as:


$$C={\left(U_{1},U_{2},\cdots ,U_{m}\right)}^{T}\times \left(D_{1},D_{2},\cdots ,D_{n}\right),$$

where *n* denotes the number of SPINs. The resulting matrix C captures the aggregate relative structural path strength through all intermediate relay neurons. Note that this measure captures the aggregate relative structural path strength through intermediate relay neurons, rather than causal influence in the sense of dynamic causal modeling (Friston et al., 2003).

### 2.9 Representative SPIN ensembles

### 2.9.1 Identifying SPIN ensemble in hemibrain

**FIGURE 4:**
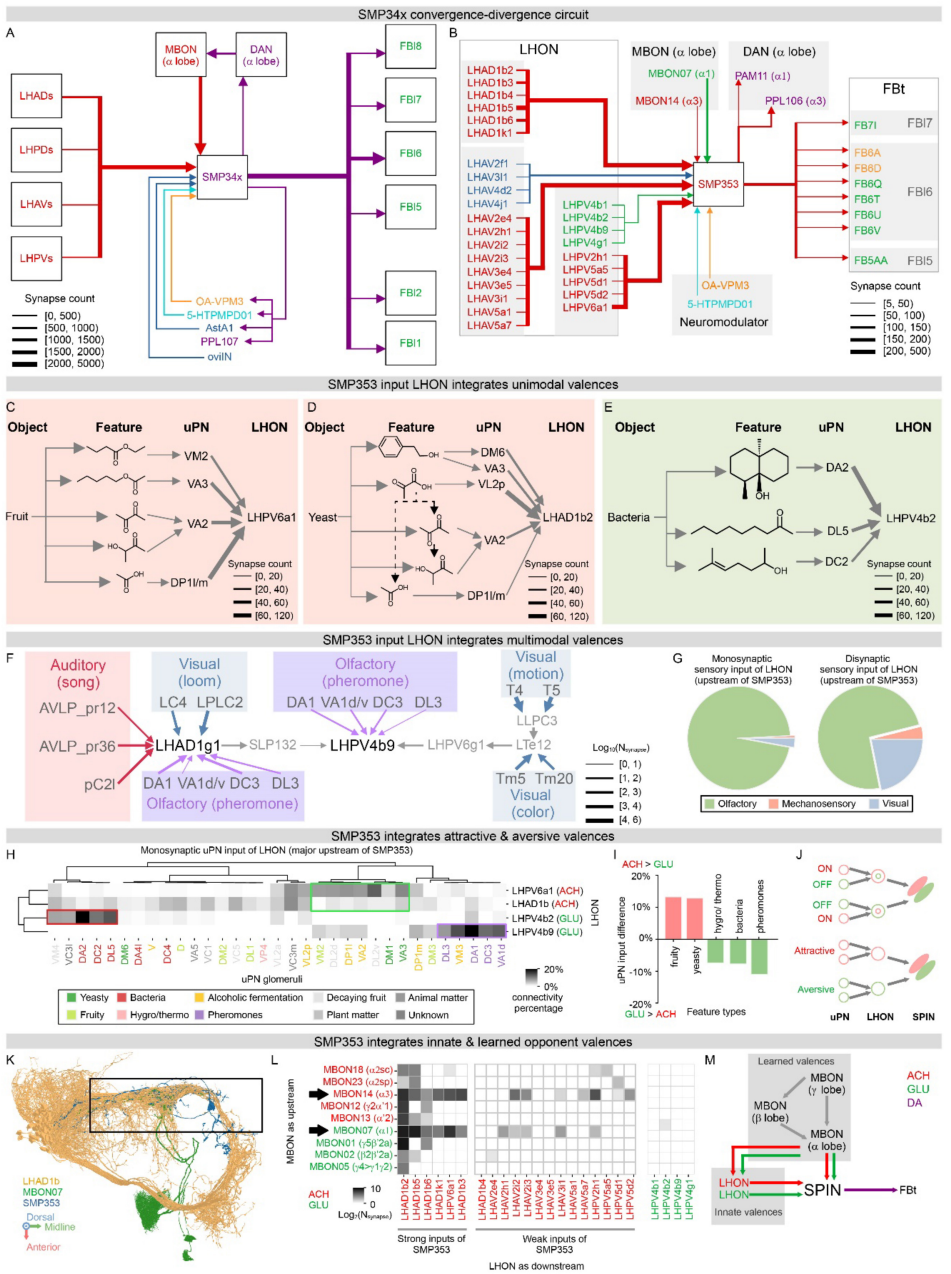
Convergence-divergence SMP353 circuit for complex valence processing. **(A,B)** Connectivity of SMP34x ensemble **(A)** and the representative SMP353 neuron **(B)** with major cell groups, including LHON, MBON, FBt, DAN, and other modulatory neurons. Line width denotes connection strengths in terms of synapse count. Line and text colors denote neurotransmitter types, where red is cholinergic, green glutamatergic, blue GABAergic, purple dopaminergic, orange octopaminergic, and cyan serotonergic. **(C–E)** Unimodal integration of chemosensory inputs from uPNs into prominent SMP353 input LHONs, including cholinergic LHPV6a1 **(C)** and LHAD1b2 **(D)** processing fruit- and yeast-related attractive cues, and glutamatergic LHPV4b2 **(E)** processing toxic bacteria related aversive cues. Each LHON integrates inputs from multiple uPNs processing diverse chemicals as sensory features, forming the chemosensory profile of natural objects. This forms a 4-stage processing system, from natural objects, to sensory features, to uPN channels, to LHON valences. Widths of lines from uPN to LHON denote synapse counts in hemibrain. **(F)** Multimodal valence integration into LHPV4b9. LHPV4b9 integrates pulse song auditory features, pheromone olfactory features, as well as color, motion, and loom visual features to signal conspecific males. Line and shade colors denote sensory modalities. Line widths denote synapse counts in FlyWire. **(G)** Modality distributions of monosynaptic (left) and disynaptic (right) sensory inputs to SMP353 upstream LHONs. **(H)** Monosynaptic connections between uPNs and prominent SMP353 input LHONs in hemibrain, including cholinergic LHPV6a1 and LHAD1b series, and glutamatergic LHPV4b2 and LHPV4b9. Hierarchical clustering of the connection matrix classified LHONs by their neurotransmitters and uPNs by their chemical features, revealing food-related attractive valences (green box) in cholinergic LHONs and bacteria and conspecific related aversive valences (red and purple boxes) in glutamatergic LHONs. We grouped uPN neurons by antennal lobe glomeruli and color-coded uPNs by reported odor features; connections are quantified by the synapse percentage of each glomerulus in the corresponding LHON. **(I)** Differences of uPN inputs between all cholinergic and glutamatergic SMP353 input LHONs in hemibrain. Food-related cues are enriched in cholinergic LHONs, while toxic bacteria, temperature, and pheromone-related aversive cues are enriched in glutamatergic LHONs. We classified uPNs by their reported odor features; positive and negative values indicate enrichment in cholinergic (red) and glutamatergic (green) LHONs, respectively. **(J)** Schematic for SPIN attractive and aversive valence integration, in analogy to integrating ON and OFF features into the receptive field in the visual circuits. Neural representations of visual receptive fields are encoded in geometric spaces, while SPIN representations are encoded in valence spaces. **(K)** SMP353 and its major input LHONs (LHAD1b series) and MBON (MBON07) processes converge together, forming a corridor (black dashed box). Other input MBONs and LHONs that are not shown, such as MBON14, also converge in the corridor. SMP353 is colored in blue, MBON07 in green, and LHAD1b series neurons in yellow, shown in the dorsal view. **(L)** Connectivity matrix from MBONs to SMP353 input LHONs in hemibrain. LHONs are grouped by strong and weak connections with SMP353 and transmitters, connections are displayed as logarithmic synapse counts, and cell types are colored by transmitters. **(M)** The MB-modulated LHON-SMP353-FBt circuit schematic, including attractive aversive innate and learned valence integration and two-layer MBON modulation.

**FIGURE 5:**
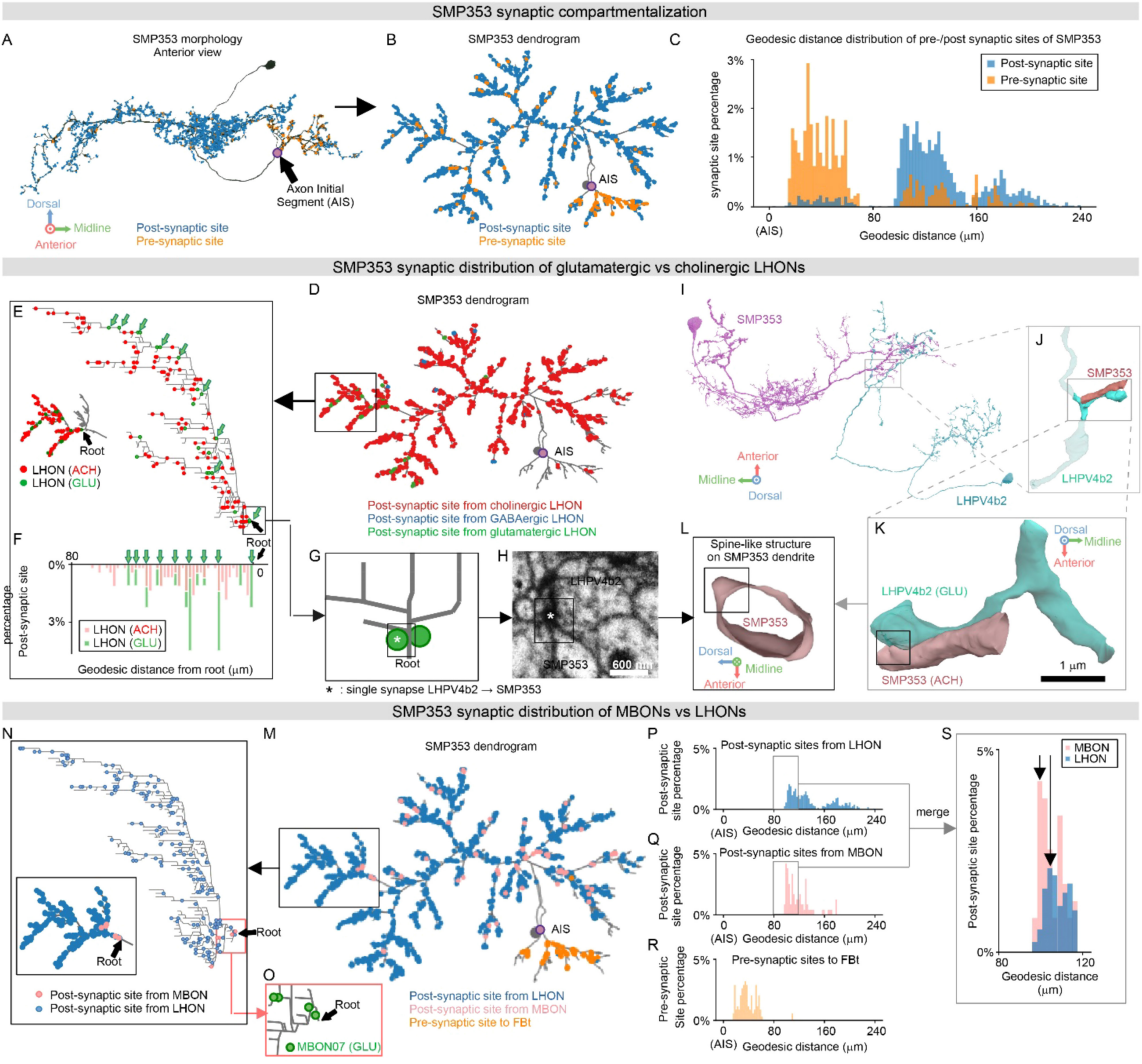
SMP353 synaptic organization. **(A,B)** Synaptic distribution of SMP353 in 3D anatomical **(A)** and 2D topological **(B)** views. Anatomical morphology shown in anterior view **(A)** is transformed into a force-directed dendrogram **(B)** via the scalable force-directed placement algorithm (see Methods). Pre- and post-synaptic sites are colored orange and blue, respectively. The putative axon initial segment (AIS) is shown in purple. **(C)** Geodesic distance distribution of all SMP353 pre- (orange) and post- (blue) synaptic sites from AIS, counted in mm. **(D)** Distribution of LHON synaptic inputs shown on SMP353 force-directed dendrogram. Post-synaptic sites from cholinergic, glutamatergic, and GABAergic LHONs are colored red, green, and blue, respectively. **(E,F)** SMP353 local branch cholinergic and glutamatergic synapse distribution. Local branch distribution of cholinergic and glutamatergic post-synaptic site from LHONs shown on SMP353 ranked dendrogram (**E**, see Methods). Post-synaptic sites from glutamatergic LHONs (green arrows) are located on the trunks of the dendrogram. Geodesic distance distribution **(F)** of post-synaptic sites from cholinergic and glutamatergic (green arrows, with one-on-one correspondence with those on dendrogram) LHONs to the root of the local branch. See Figures 6L,M for comparison. **(G,H)** A single glutamatergic synapse from LHPV4b2 to SMP353 located near the root of the local branch in panel **(E)**, shown on ranked dendrogram **(G)**, and on EM image **(H)**. Scale bar: 600 nm. **(I–L)** 3D reconstruction of the single glutamatergic synapse in panels **(G,H)**, shown on whole cell **(I)**, primary neurite **(J)**, single pre-synaptic bouton **(K)**, and post-synaptic structure highlighting spine-like protrusion **(L)**. Scale bar in panel **(K)**: 1000 nm. **(M–O)** Organized synaptic distribution of LHON and MBON inputs to SMP353 and FBt outputs, shown on force-directed dendrogram **(M)**. Post-synaptic sites from LHONs and MBONs are colored in blue and pink, respectively, pre-synaptic sites to FBt neurons are colored in orange, and putative AIS is shown in purple. MBON synapses are distributed more proximally than LHON synapses on a representative local branch, as shown on the ranked dendrogram **(N)**, especially glutamatergic synapses from MBON07 **(O)**. See Figures 6N–P for comparison. **(P–S)** SMP353 geodesic distance distribution of LHON **(P)** and MBON **(Q)** inputs and FBt **(R)** outputs, measured from synaptic sites to AIS in mm. Overlaid distributions **(S)** of MBONs and LHONs show that MBON input synapses are located more proximally than LHON synapses. Arrows highlight peaks of MBON and LHON distributions. See Figures 6Q–S for comparison.

**FIGURE 6:**
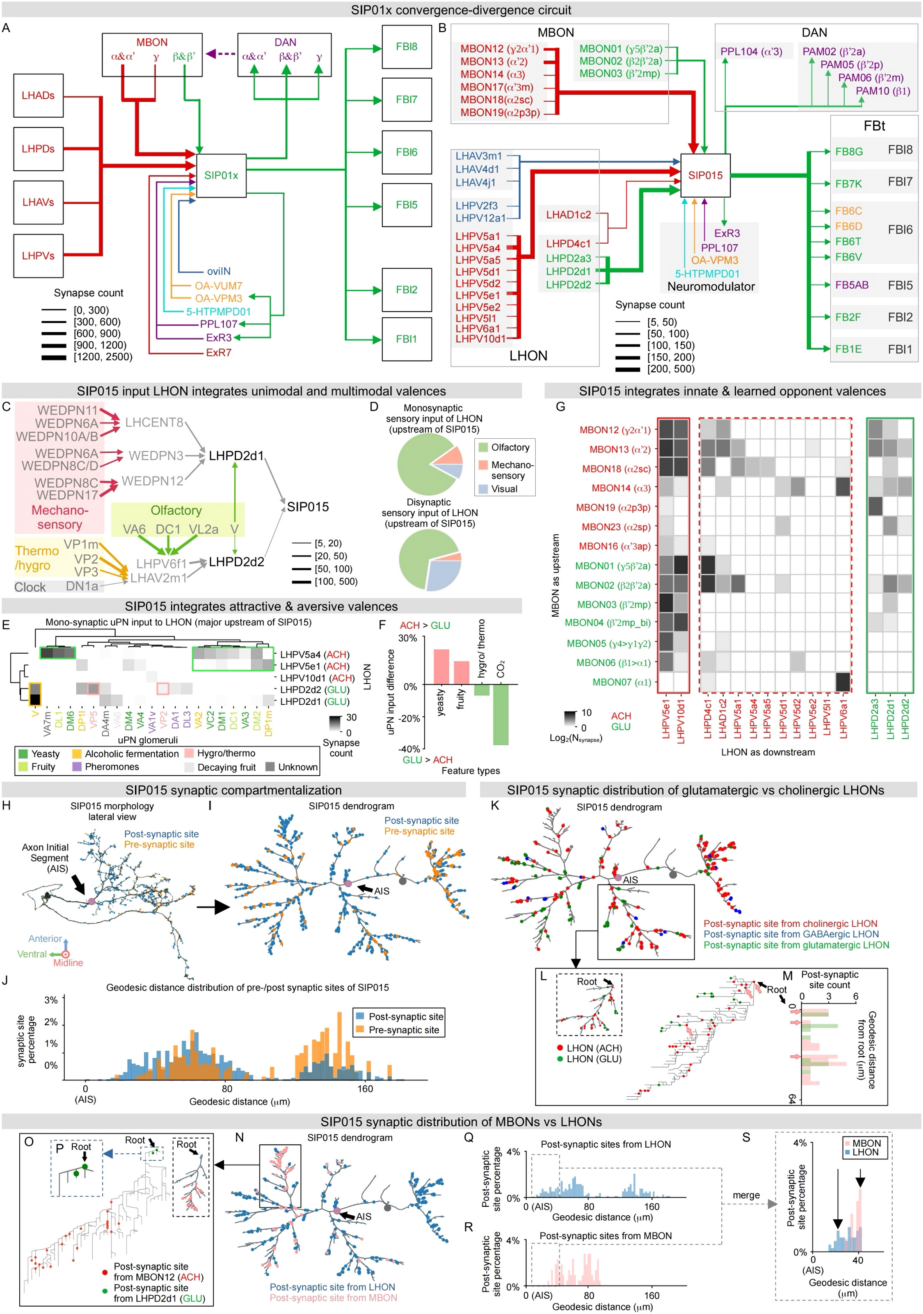
Convergence-divergence circuit and synaptic organization in SIP015 for complex valence processing. **(A,B)** Connectivity of SIP01x ensemble **(A)** and the representative SIP015 neuron type **(B)** with major cell groups, including LHON, MBON, FBt, DAN, and other modulatory neurons. Line width denotes connection strengths in terms of synapse count. Line and text colors denote neurotransmitter types, where red is cholinergic, green glutamatergic, blue GABAergic, purple dopaminergic, orange octopaminergic, and cyan serotonergic. **(C)** Multimodal valence integration into LHPD2d1 and LHPD2d2, two primary glutamatergic LHON inputs of SIP015. LHPD2d1 broadly integrates mechanosensory inputs via a hierarchical convergent circuit. LHPD2d2 integrates thermal, hygral, olfactory, and circadian multimodal inputs. Thermo- and hygro- inputs include cold, hot, and humid features. Olfactory inputs include uPNs processing multiple aversive odors and carbon dioxide. These aversive thermal, hygral, and olfactory inputs, together with circadian inputs, signal aversive conditions. Line and shade colors denote sensory modalities. Line widths denote synapse counts. **(D)** Modality distributions of monosynaptic (upper) and disynaptic (lower) sensory inputs to SIP015 upstream LHONs. **(E)** Monosynaptic connections between uPNs and prominent SIP015 input LHONs, including cholinergic LHPVa4, LHPV5e1, and LHPV10d1, and glutamatergic LHPD2d1 and LHPD2d2. Hierarchical clustering classified LHONs by their neurotransmitters and uPNs by their chemical features, revealing food-related attractive valences (green boxes) in cholinergic LHONs, carbon dioxide, and thermosensory (yellow and pink boxes) inputs in glutamatergic LHONs. We grouped uPN neurons by antennal lobe glomeruli and color-coded uPNs by reported odor features; connections are quantified by the synapse percentage of each glomerulus in the corresponding LHON (see Methods). **(F)** Differences of uPN inputs between all cholinergic and glutamatergic SIP015 input LHONs. Food-related cues are enriched in cholinergic LHONs, while temperature and CO_2_-related aversive cues are enriched in glutamatergic LHONs. We classified uPNs by their reported odor features, positive and negative values indicate enrichment in cholinergic (red) and glutamatergic (green) LHONs, respectively. **(G)** Connectivity matrix from MBONs to SIP015 input LHONs. LHONs are grouped by strong and weak connections with SIP015 and transmitters, connections are displayed as logarithmic synapse counts, and cell types are colored by transmitters (see Methods). **(H,I)** Synaptic distribution of a representative SIP015 neuron in 3D anatomical **(H)** and 2D topological **(I)** views. Anatomical morphology shown in lateral view **(H)** is transformed into a force-directed dendrogram **(I)** via the scalable force-directed placement algorithm (see Methods). Pre- and post-synaptic sites are colored in orange and blue, respectively. Putative AIS is shown in purple. **(J)** Geodesic distance distribution of all SIP015 pre- (orange) and post- (blue) synaptic sites from AIS. **(K)** Distribution of LHON synaptic inputs shown on SIP015 force-directed dendrogram. Post-synaptic sites from cholinergic, glutamatergic, and GABAergic LHONs are colored red, green, and blue, respectively. **(L,M)** SIP015 local branch cholinergic and glutamatergic synapse distribution. Local branch distribution of cholinergic and glutamatergic post-synaptic sites from LHONs is shown on the SIP015 ranked dendrogram (**L**, see Methods). Post-synaptic sites from cholinergic LHONs (pink arrows) are located more proximally on the trunks of the dendrogram. Geodesic distance distribution **(M)** of post-synaptic sites from glutamatergic and cholinergic (pink arrows, with one-on-one correspondence with those on ranked dendrogram) LHONs to the root of the local branch. See Figures 5E,F for comparison. **(N–P)** Organized synaptic distribution of LHON and MBON inputs to SIP015. Post-synaptic sites from LHONs and MBONs are colored in blue and pink, respectively, and AIS is shown in purple on the force-directed dendrogram **(N)**. LHON synapses are distributed more proximally than MBON synapses on a representative local branch, as shown on the ranked dendrogram **(O,P)**, especially glutamatergic synapses from LHPD2d1 versus cholinergic synapses from MBON12. See Figures 5M,N for comparison. **(Q–S)** SIP015 geodesic distance distribution of LHON **(Q)** and MBON **(R)** inputs, measured from synapses to AIS. Overlaid distributions **(S)** of MBONs and LHONs show that LHON input synapses are located more proximally than MBON synapses. Arrows highlight local peaks of MBON and LHON distributions. See Figures 5P–S for comparison.

Neurons similar in morphology and connectivity are more likely to be developmentally and functionally related, thus we identified ensembles based on morphology and connectivity similarity. To filter clusters defined by connectivity further with morphology, we estimated the morphological similarity for each SPIN cluster. We first used the NBLAST algorithm (Costa et al., 2016) (see Geometry session) to quantify the morphological similarity of all SPIN pairs and normalized the similarity matrix of all SPINs (Supplementary Figure 5C). Next, for clusters with the same regional connectivity preference (e.g., LH-related SPINs), we used a *t*-test to determine if the mean intra-cluster and inter-cluster similarity scores of a specific cluster are significantly different. SPIN clusters with significantly different morphological similarities are selected as ensembles, SMP34x (Figures 4, 5) and SIP01x (Figure 6).

### 2.9.2 Matching SPIN ensemble in FlyWire

We matched all ensembles between hemibrain and FlyWire based on cross-brain morphological similarity. We first aligned all SPINs in both hemibrain and FlyWire datasets to the same standard brain template (JRC2018F) (Bogovic et al., 2020). For FlyWire SPINs, we then mirrored the SPINs in left hemisphere to the right hemisphere in order to compare the morphological similarity together. Next, we used the NBLAST algorithm (Costa et al., 2016) to calculate the pairwise similarity score between all SPINs from both datasets (see Geometry session). After that, we calculated the similarity score distribution for each ensemble and for all SPINs. We took the (*mean*−*sd*) of similarity score for each ensemble in hemibrain as the morphological threshold. FlyWire SPINs with intra-cluster similarity scores higher than the morphological threshold defined in hemibrain, (*mean*−*sd*), were considered the same ensemble as in hemibrain.

### 2.9.3 Comparison of the connectivity strength of major cell types with SPIN ensembles

To calculate the average connectivity percentage of all neurons in each SPIN ensemble with each major cell type in LH, MB, and FB, we used the number of synapses connected with major cell types divided by the numbers of all synapses of each SPIN, and then averaged across SPINs, for pre-synaptic and post-synaptic sites. Only major cell types whose average connectivity percentages with SPIN ensemble are more than 0.1% in hemibrain or FlyWire are shown. We also calculated the percentage difference between the two datasets by subtracting the percentage in FlyWire from that in hemibrain (Supplementary Figures 7F, 9D).

### 2.10 Geometry

We used the NBLAST algorithm (Costa et al., 2016) to calculate the pair-wise morphological similarity score among all SPINs. NBLAST works by decomposing neurons into point and tangent vector representations. One neuron is designated the query and the other the target. For each query segment (defined by a midpoint and tangent vector) the nearest neighbor (using straight-forward Euclidean distance) is identified in the target neuron. A score for the segment pair is calculated as a function of two measurements: *d*, the distance between the matched segments (indexed by *i*), and $\left|\vec{u_{i}}\cdot \vec{v_{i}}\vee \right|$, the absolute dot product of the two tangent vectors; the absolute dot product is used because the orientation of the tangent vectors has no meaning in our data representation. The scores are then summed over each segment pair to give a raw score, *S*:


$$S\,\left(q\,u\,e\,r\,y,t\,a\,r\,g\,e\,t\right)=\sum_{i=1}^{n}f\,\left(d_{i},\left|\vec{u_{i}}\cdot \vec{v_{i}}\right|\right).$$

To determine a suitable *f*, we developed an approach inspired by the scoring system of the BLAST algorithm. For each segment pair we defined the score as the log probability ratio:


$$f=l\,o\,g_{2}\,\frac{p_{m\,a\,t\,c\,h}}{p_{r\,a\,n\,d}},$$

the probability that the segment pair was derived from a pair of neurons of the same type, versus a pair of unrelated neurons. As NBLAST (Costa et al., 2016) is a point- and vector-based searching algorithm, to prevent the node number of neuron skeletons from influencing the calculation of similarity scores, we first aligned the node distribution of the SPIN skeleton in hemibrain and FlyWire. We downsampled FlyWire and hemibrain SPINs by 10 and 2 times, respectively to align the two node-count distribution of SPIN skeleton. Next, considering that the SPINs in FlyWire are located in both hemispheres, while the SPINs in hemibrain are located in the right hemisphere only, we mirrored all SPIN in left hemisphere of FlyWire to the right side so that we can compare their morphological similarity with SPINs in hemibrain. To compare the SPINs from the two datasets, we then aligned these neurons to the same brain template JRC2018F (Bogovic et al., 2020). After that, we transformed the skeleton tree of SPIN to a point and tangent vector representation (using 5 nearest neighbors to calculate tangent vector) of neurons (dotdrops) to calculate the NBLAST pairwise similarity score among all SPINs, and to obtain the pairwise similarity score matrix. We then diagonalized the similarity score matrix and normalized it by line. We performed hierarchical clustering on the matrix to anatomically divide SPINs into 40 clusters (Supplementary Figure 5C). We also counted the cell numbers of each cell type of SPIN and central complex neurons in hemibrain and calculated the cell number distribution across cell types (Figures 2M,N).

### 2.11 Subcellular topology

Skeletons in hemibrain and FlyWire are saved as hierarchical trees. As such they can be visualized as graph-like dendrograms. In this paper, we used the scalable force-directed placement (sfdp) algorithm (Hu, 2006) and the hierarchical layout algorithm (Gansner et al., 1993) to embed neuron skeletons onto 2D dendrograms. Notice that before embedding, we usually rerooted the neurons to the soma to better visualize the topological relationship of the branches.

#### 2.11.1 Force-directed dendrogram

We transformed the skeleton tree of the complete neuron by the sfdp algorithm (Hu, 2006) (e.g., Figures 5B, 6I). Based on the spring-electrical model (Fruchterman and Reingold, 1991), sfdp is a fast, multilevel, force-directed algorithm that efficiently lays out large graphs (Hu, 2006). This model assigned two forces between vertices in the graph, and finding the optimal layout by minimizing the energy of the graph. The repulsive force, which exists between any two vertices, is inversely proportional to the distance between them. Meanwhile, the attractive force exists only between neighboring vertices, and is proportional to the square of the distance. The combined force of all vertices is therefore the energy of the graph. We optimized the graph layout by minimizing the energy. The energy of the spring-electrical model can be minimized iteratively by moving the vertices along the direction of forces exerted on them. And we measured the progress by the decrease in system energy.

#### 2.11.2 Ranked dendrogram

We transformed the skeleton tree of selected branches to a ranked dendrogram by a hierarchical layout algorithm (Gansner et al., 1993) in Figures 5E,N, 6L,O. We first assigned a specific vertical level (rank) to each node to organize the layout from top to bottom. If an edge spans multiple levels, we split it by adding temporary “virtual nodes” (like stepping stones) to break long connections into shorter, single-level segments. Nodes on the same vertical level were rearranged horizontally to prevent overlapping edges and keep connections visually clear. Finally, we adjusted the horizontal positions of all nodes to minimize edge lengths.

#### 2.11.3 Locate synapse position on dendrogram

For the visualization of the synaptic sites on the dendrogram, we calculated the nearest point on the original skeleton tree for each synaptic site detected and reassigned the position of the nearest point to the synaptic site. We visualized the synaptic sites on the dendrogram based on the reassigned positions.

### 2.12 Neurotransmitter prediction

We combined the LM and EM data (Chiang et al., 2011; Aso et al., 2014a; Dolan et al., 2019; Li et al., 2020; Hulse et al., 2021; Eckstein et al., 2024) to predict the neurotransmitters of neurons. For the prediction for SPINs, we mainly referred to the “top” neurotransmitter prediction of hemibrain and FlyWire. If the neurons of the same SPIN type have different transmitter prediction results, we preferentially select the neurotransmitter with a higher proportion as the prediction result. Moreover, if the prediction result differs from the LM data, we preferred to label the neuron with LM results.

#### 2.12.1 Neurotransmitter comparison between hemibrain and FlyWire

We compared the transmitter percentages of the whole brain, all SPINs (Figures 2H,I), SMP34x (Supplementary Figure 7E), and SIP01x (Supplementary Figure 9C) in hemibrain and Flywire based on their neurotransmitter predictions. For SMP34x and SIP01x neurons, we calculated the average percentage of pre-synaptic sites that are predicted to express specific kinds of transmitter (including acetylcholine, glutamate, GABA, dopamine, serotonin, and octopamine) of all neurons in both hemibrain and FlyWire, and generated the heatmaps with the contrast denoting the average percentage of each neurotransmitter.

### 2.13 Single-cell sequencing analysis

Expression analyses were performed by using the digital expression matrix of the existing sequencing datasets with preprocessing (Croset et al., 2018; Davie et al., 2018; Janssens et al., 2022; Li et al., 2022). We considered glutamate receptor genes for GluClα, GluRIA, GluRIB, and GluRIIA. Cells containing more than 0 UMIs of the marker gene were considered. There were a small number of non-neuronal cells in the original dataset, including astrocytes, fat body cells, and other glial cells, which may influence the results to some extent.

### 2.14 Code and data information

All analyses were done in the Python3 environment. Analysis and visualization of connectivity data was performed by *numpy*, *pandas*, and *matplotlib* packages in python. UMAP downsampling analysis was performed by *umap* package^1^ in python. Analysis and visualization of morphological data were performed based on the *NAVis* package^2^. Motif analysis in Figures 2B–G was done by *dotmotif* package (Matelsky et al., 2021)^3^. EM raw images and segmentation masks in 2D views (Figure 5H, Supplementary Figures 8A–C) were visualized by Neuroglancer^4^. The 3D reconstruction of neurons (Figures 5I–L) was generated by Blender. The structures of chemicals in Figures 4C–E were generated by MolView^5^.

## 3 Results

### 3.1 Intrinsic neurons of the superior protocerebrum

To identify regions connected with LH, MB, and CX, we focused on superior neuropils amid MB, LH, and CX, occupying the superiormost parts of the brain (Figures 1A,B), including superior medial protocerebrum (SMP), superior intermediate protocerebrum (SIP), and superior lateral protocerebrum (SLP) (Supplementary Figure 1A; Ito et al., 2014). The crepine (CRE) surrounding the MB medial lobe forms a concrete network with superior neuropils (Supplementary Figures 1B,C, also see Methods). This study refers to them as superior protocerebrum (SP). To improve robustness, we examined both the hemibrain and the FAFB/FlyWire datasets throughout this study.

To study SP circuits, we reasoned that its intrinsic neurons are likely central to its core functions. Thus, we systematically identified SP intrinsic neurons. We statistically filtered well-traced SP neurons via synaptic distribution (Supplementary Figures 1D–H, also see Methods). By examining pre- and post-synaptic site distribution of well-traced SP neurons in unilateral SP, we identified a neuron cluster with over 85% of their synapses within SP (Figures 1C,D, green squares) as SP intrinsic neurons (SPIN) in hemibrain and FlyWire. After further statistical filtering via synaptic distribution (see Methods), the final counts for the total SPINs are 1411 in the right hemisphere in hemibrain, and 2844 bilaterally in FlyWire (Figures 1E,F), which are highly consistent. We focused on SPINs in this study. Interestingly, well-traced SP neurons, with arborizations both in and out of SP, have more pre-synaptic than post-synaptic sites in SP (Figures 1C,D, orange lines are polynomial fittings, see Methods). Alternatively, SP neurons have more post-synaptic sites outside SP. Given that pre-synaptic and post-synaptic sites correspond to synaptic outputs and inputs, respectively, the observed asymmetry suggests that the SP region receives a greater number of inputs via SP neurons from outside than it provides outputs. This leads to the hypothesis that SP may play a role in information integration. To further test the information integration hypothesis, we next examined the SPIN-connected circuits, on the level of brain regions, neuronal cell groups, and single neurons across the brain.

### 3.2 Brain-wide connectivity of SPINs linking LH, MB, and FB

To explore the brain-wide connectivity of SPINs, we examined at the brain region, cell type, and single-cell levels. We first traced their major inputs and outputs by brain regions (Supplementary Figures 1I,J). We analyzed the post-synaptic site locations of SPIN upstream neurons and the pre-synaptic site locations of SPIN downstream neurons. Apart from SP, the primary input regions are LH and MB, whereas the top output region is FB, consistent between hemibrain and FlyWire. To further pinpoint SPIN connectivity with cell type resolution, we then examined the major input and output cell groups of MB, LH, and FB. These include dopaminergic neuron (abbreviated as DAN) and MB output neuron (MBON) in MB, LH centrifugal neuron (LHCENT) and LH output neuron (LHON) in LH, FB tangential neuron (FBt) and FB columnar neuron (FBc) in FB, as well as modulatory neurons (Figures 1G–J). The numbers of synaptic sites are in the order of tens of thousands, with about twice as many SPIN post-synaptic as pre-synaptic sites (Figures 1G–J). These results are consistent between hemibrain and FlyWire, supporting prominent connections for information integration into SPINs. In both hemibrain and FlyWire, we found the most abundant upstream and downstream synaptic partners of SPINs to be LHON and FBt neurons, respectively (Figures 1G–J, arrows), suggesting LHON-SPIN-FBt connectivity as the dominant direction of information flow. To gain single-cell resolution, given the complexity of connectome data, we performed dimensionality reduction on the complete connectivity of SPINs with all neurons in the connectome to map whole-brain single-cell connectivity of all SPINs holistically. Using UMAP, we generated a SPIN brain-wide connectivity atlas (Figures 1K–N, Supplementary Video 1, see Methods). To examine whether specific cell populations are clustered, we classified SPINs via connectivity using density-based clustering (Figures 1K,M, see Methods). We indeed found that each arm of the pentagram-shaped embedding is populated by cells with similar connectivity. Wondering how these cell classes are related to major input and output regions, we quantified the ratios of SPIN connections with MB, LH, and FB to color-code the SPINs on the atlas (Figures 1L,N). Indeed, MB- and LH-related SPINs occupy discrete arms, while FB-related SPINs are located in the middle, adjacent to all arms (Figure 1L, Supplementary Video 2). Because proximity in a UMAP embedding reflects similarity in the high-dimensional data, such an organization suggests that FB may serve as a hub of the SPIN circuit, consistent with the information integration hypothesis. In the FlyWire dataset, we observed distribution patterns similar to those in the hemibrain atlas (Figure 1O). With a single-cell connectome, these results support information integration from MB and LH into FB.

Collectively, our connectivity analyses at the brain region, cell type, and single-cell levels reveal the LH/MB-SP-FB pathway as the dominant route of information flow. Wondering about the topological structure of SP circuit linking MB, LH, and FB, we proposed three circuit models (Figure 2A): (1) a labeled-line model, in which specific SPINs process dedicated inputs and outputs to form parallel channels; (2) a mixed model, in which all SPINs process all inputs and outputs, yielding an all-to-all fully-connected network; and (3) an integration model, inspired by the information integration nature of SP, wherein specific SPINs integrate multiple dedicated inputs and outputs to establish a convergence-divergence network.

To distinguish between these models, we calculated the Shannon entropy (Shannon, 1948; Betzel et al., 2024; Moon et al., 2026) of the input and output connectivity distributions for individual SPINs (Supplementary Figure 3). The observed entropies were significantly above zero yet well below the theoretical maximum, rejecting both the labeled-line (Supplementary Figures 3D,G,J,M) and the mixed (Supplementary Figures 3E,H,K,N) model, respectively. Furthermore, to determine whether SPIN connectivity exhibits non-random topological structures, we evaluated a random wiring model generated by shuffling the network connectivity. The observed entropy deviated significantly from that of the shuffled networks (Supplementary Figures 3F,I,L,O), effectively ruling out the random wiring model. Ultimately, the observed entropy profile, moderate mean with high variance across SPINs (distributions in Supplementary Figures 3D,G,J,M), confirms that SPINs exhibit structured, non-uniform connectivity with dedicated input and output partners. This observed architecture of the SPIN circuit strongly aligns with the preceding cell-class analyses. Under a strict labeled-line model, individual SPINs would connect exclusively to dedicated neurons, resulting in discrete cell classes. Conversely, a mixed model dictates that SPINs would innervate completely overlapping neuronal populations, leading to a smooth, uniform gradient of cell classes. Instead, because the circuit exhibits structured, non-random connectivity, the resulting SPIN cell-class fingerprints form a complex gradient characterized by cell-class-specific slopes that reflect their precise connectivity profiles.

### 3.3 Circuit motifs as combinatorial building blocks of SPIN networks

Complex neural circuits can be decomposed into simpler fundamental units (Luo, 2021). Because networks can be modeled as directed graphs, with recurring motifs serving as fundamental units (Milo et al., 2002; Bullmore and Sporns, 2009; Ma et al., 2009), we asked if complex SPIN circuits are combinatorically assembled from generic motifs.

Given that Shannon entropy analyses indicated structured SPIN connectivity, we next sought to identify the specific network architecture by analyzing triplet motifs, which served as an alternative validation of SPIN network models (Figure 2A). We posited that a strict labeled-line model would yield very few triplet motifs. Instead, we found a high frequency of triplet motifs (Figures 2B–G), arguing against labeled-line architecture. To distinguish between the remaining two models, we examined the balance of motif types: a mixed model should exhibit roughly equal numbers of convergence and divergence motifs. However, we observed unequal frequencies in both hemibrain and FlyWire datasets, favoring the integration model. Finally, we tested a key prediction of the integration model: that convergence and divergence motifs should be enriched in input and output circuits, respectively. Our data confirmed this distribution in both datasets. Together, these analyses indicate that the integration model, rather than the labeled-line or mixed models, best describes SPIN circuitry.

Convergence and divergence motifs can facilitate information integration and broadcasting (Jeanne and Wilson, 2015; Luo, 2021), and our analyses indicate that these are key functions of SP input and output circuits, respectively (Figures 2B–G, Supplementary Figure 3). Interestingly, the convergence-divergence network is analogous to an autoencoder network (Lecun and Soulie Fogelman, 1987; Kramer, 1991; Goodfellow et al., 2014), enabling the processing of complex inputs to generate complex outputs via sequential dimensionality reduction and expansion.

### 3.4 SPINs are primarily inhibitory or modulatory

Neural circuits with identical connectivity can execute profoundly different functions depending on their constituent cell types and neurotransmitter profiles. To further probe how the SP convergence network integrates information, we posited that this integration occurs via either summation or subtraction. Ultimately, the neurotransmitter identities of the constituent neurons largely determine whether a triple convergence motif is configured for additive or subtractive computation.

We examined the neurotransmitter profiles of all SPINs based on neurotransmitter predictions (Eckstein et al., 2024). Compared to the whole brain, the proportion of glutamatergic and cholinergic SPINs are significantly enriched and depleted, respectively (Figures 2H,I). Glutamate can act as an inhibitory transmitter via glutamate-gated chloride channel GluClα (Liu and Wilson, 2013). However, ionotropic glutamate receptor subunits homologous to mammalian AMPA and NMDA receptors are also expressed in *Drosophila*, indicating excitatory transmission. To estimate the extent of glutamate-mediated inhibition in the central brain, we reasoned that the expression frequency of GluClα serves as a proxy for the probability of inhibitory glutamatergic transmission. Analysis of published central brain single-cell sequencing data (Croset et al., 2018; Davie et al., 2018; Janssens et al., 2022; Li et al., 2022) revealed that GluClα is indeed the dominant glutamate receptor (Li et al., 2022; Figure 2L), suggesting that glutamatergic SPINs predominantly act as inhibitory neurons. Consequently, by combining glutamatergic and GABAergic populations, over half of SPINs are inhibitory. Furthermore, inhibitory and modulatory neurons together constitute three-quarters of SPINs in hemibrain, posing constraints on SP function. To determine whether SPINs with specific neurotransmitters profiles exhibit regional clustering, we mapped these cell types onto a brain-wide connectivity atlas (Figures 2J,K, Supplementary Figures 5A,B, and Supplementary Video 3). Dopaminergic SPINs are clustered, primarily comprising FB- and MB-related SPINs. Conversely, cholinergic and glutamatergic SPINs are broadly distributed with a bias toward LH- and MB-related SPINs (Supplementary Figures 5A,B). This distribution suggests a trend wherein the convergence-divergence circuit funnels widespread excitatory and inhibitory inputs to drive focused dopaminergic outputs, an organization similar to mammalian dopaminergic circuits (Hu, 2016; Watabe-Uchida et al., 2017).

### 3.5 SPIN cell types are composed of single neurons or small ensembles

Neural information can be encoded either by ensembles of similar neurons or by individual neurons. Neuron ensembles facilitate population coding to reliably represent complex variables (Vyas et al., 2020), such as head direction tracked by central complex EPG compass neurons (Seelig and Jayaraman, 2015). Conversely, individual neurons efficiently encode simpler signals, as seen in the giant fiber neurons mediating escape choices (Asinof and Card, 2024). Because neurons are metabolically expensive, the brain likely minimizes the number of neurons allocated to each function; thus, the size of a neuron population reflects the complexity of the information it processes. Consequently, single neurons or small ensembles are more efficient at encoding low-dimensional features than larger ones. Because the convergence-divergence topology of the SP network functions much like the bottleneck layer of an autoencoder, compressing data into a lower dimension, we hypothesized that SPINs are composed of single neurons and small ensembles optimized for encoding low-dimensional information. To examine the cell numbers across SPIN cell types, we first examined cell types defined in hemibrain and FlyWire. We reasoned that cells of the same type should be similar in morphology and connectivity. To check morphology, we quantified the morphological similarity between SPINs using the NBLAST algorithm (Costa et al., 2016), and indeed found that neurons within each ensemble exhibit homogeneous morphology by clustering (Supplementary Figure 5C). Wondering whether neurons with similar morphology also assume similar connectivity, we mapped SPINs on whole-brain connectivity atlas based on morphology (Supplementary Figures 5D,E), and found that neurons with similar morphology are clustered on the atlas, similar to connectivity, supporting cell type definition in hemibrain and FlyWire, further supporting consistency between datasets. We then examined the distribution of cell numbers across all SPIN cell types. Indeed, most SPIN types contain one or two neurons on each hemisphere, while only nine types of SPINs contain more than six neurons (Figures 2M,N, Supplementary Figure 5F). Six of the nine types are inhibitory, and the remaining three are dopaminergic, consistent with the inhibitory or modulatory neurochemical signature of all SPINs (Figures 2H,I). Together with the neurochemical distribution, SPINs are composed of glutamatergic populations and cholinergic single neurons as inputs, and dopaminergic populations as outputs. These single neuron or small ensemble results indicate low-dimensional information in SPINs, as elaborated later.

Based on our connectivity, motif, and cell-type analyses, we propose a SPIN circuit architecture model (Figure 2O). SPINs receive inputs from major output cell groups LHON, MBON, and FBc and project to key input cell groups LHCENT, DAN, and FBt. Notable differences in connection strengths suggest that the LHON/MBON-SPIN-FBt axis serves as the dominant pathway for information flow (Figure 2O). While convergence and divergence network motifs characterize SPIN input and output circuits, reinforcing an integration model, it remains uncertain whether the SPIN circuit also employs a mixed model or a labeled-line model. Furthermore, despite receiving widespread inputs, it is unclear whether these inputs are driven by a single sensory modality or exhibit multimodal characteristics. We investigated these questions by analyzing specific SPIN input and output regions.

### 3.6 SPINs integrate multimodal convergent inputs

The lateral horn (LH) receives diverse sensory inputs to mediate innate behaviors (Dolan et al., 2019; Frechter et al., 2019; Bates et al., 2020; Schlegel et al., 2021). Connectivity analysis revealed that most SPINs receive substantial synaptic inputs from LHONs (Figures 1G,I) while sending relatively less output back to LHONs and LHCENTs (Figures 1H,J), positioning LH upstream of SPINs (Figure 3A). To further probe the LH-SPIN network, we traced the multi-layer connectivity with major cell groups of SPINs with strong LH preferences (see Methods). LHONs serve as the primary direct inputs, followed by MBONs, while FBt neurons emerge as the principal outputs via two SPIN layers (Figure 3B, arrows), consistent between hemibrain and FlyWire. To pinpoint the topology of LHON to SPIN circuit, we classified SPINs via hierarchical clustering of the SPIN-LH connection matrix (see Methods). Each SPIN cluster samples feedforward input from many dedicated LHONs (Figures 3C,D). This LHON to SPIN mapping is largely exclusive: each LHON targets a specific SPIN cluster rather than the entire population. Thus, multiple LHONs converge onto dedicated SPINs in a many-to-one relation, ruling out a mixed model and supporting a convergence integration model. We then asked whether SPINs directly interact with sensory or motor neurons. A survey of connections from olfactory (OPNs), mechanosensory (WEDPNs), and visual (VPNs) projection neurons, alongside descending motor neurons (DNs) (Wu et al., 2016; Namiki et al., 2018; Bates et al., 2020), revealed clear direct inputs only from OPNs (Supplementary Figures 6A,B, upper panels). These OPN-recipient SPINs integrate both OPN and LHON inputs before projecting to FBt neurons via two SPIN layers (Supplementary Figure 6D). This pattern parallels the LH inputs, suggesting a functional coupling between OPN and LHON signals. Most SPIN-targeting OPNs are multiglomerular (mPN) rather than uniglomerular (uPN) (Supplementary Figure 6E; Bates et al., 2020). These mPNs pool information across multiple antennal lobe glomeruli (Tanaka et al., 2012; Bates et al., 2020; Schlegel et al., 2021), with many mediating thermosensory and hygrosensory processing. To assess the mPN-SPIN network topology, we clustered SPINs based on their PN connectivity. Unlike the convergent LHON-SPIN network (Figure 3D), each OPN-SPIN cluster is exclusively innervated by only one or two specific mPNs (Supplementary Figure 6E), supporting a labeled-line model.

Olfactory projection neuron inputs are considerably weaker than those from LH and MB (Supplementary Figure 6D, compare with Figures 3B,M), indicating that higher-order neurons dominate SPIN inputs while direct sensory connections contribute minimally. Given this lack of direct sensory drive, we hypothesized that SPINs acquire sensory information indirectly via higher-order networks. This is highly plausible: the lateral horn (LH), traditionally viewed as an olfactory center, is now recognized as a multisensory hub processing visual, mechanosensory, thermosensory, and hygrosensory inputs (Fişek and Wilson, 2014; Dolan et al., 2019; Frechter et al., 2019; Bates et al., 2020; Marin et al., 2020; Schlegel et al., 2021). Furthermore, the AVLP, a higher-order region rich in visual, auditory, and somatosensory information (Wu et al., 2016; Tsubouchi et al., 2017; Baker et al., 2022) funnels vital input to SP (Supplementary Figures 1I,J). To systematically map sensory modalities, we traced visual, olfactory, and mechanosensory inputs to SPINs across 0–5 intermediate layers. Most sensory inputs arrive via circuits with 1–3 intermediate layers, peaking at 2 layers, in both hemibrain and FlyWire (Supplementary Figures 6F,G). Focusing on disynaptic and trisynaptic circuits (Figures 3E–G), we found that SPINs receive massive multimodal drives, especially via trisynaptic pathways (Figure 3F, Supplementary Figure 6G). Mapping the anatomical origins of the first-layer neurons in both disynaptic and trisynaptic circuits (Figure 3G) revealed contributions from nearly every brain region, demonstrating that SPINs integrate brain-wide sensory inputs.

Together, SPINs integrate deeply processed multimodal inputs from diverse sources but barely from direct sensory inputs. This pronounced detachment from early sensorimotor pathways contrast sharply with *C. elegans* connectome, where most neurons are innervated by primary sensory and motor neurons (Cook et al., 2019), implying that SPINs mediate evolutionary advanced, higher-order computations. Situated downstream of deep multimodal circuits, SPINs are well poised to compute higher-order abstractions from sensory streams rather than participating in basic sensory feature detection. Valence is a strong candidate: LH is a known innate valence center (Dolan et al., 2019; Frechter et al., 2019; Bates et al., 2020; Schlegel et al., 2021), MB governs learned valence, while FB uses valence information, as discussed below.

### 3.7 SPINs send divergent outputs to FB

The fan-shaped body (FB) of the central complex (CX) is implicated in goal-directed action selection informed by valence (Cayco-Gajic and Silver, 2019; Shiozaki et al., 2020; Matheson et al., 2022; Ishida et al., 2026; Mussells Pires et al., 2024; Westeinde et al., 2024). FB contains columnar and tangential neurons (FBc and FBt) (Hulse et al., 2021). To further analyze the connectivity of the SP-FB network, we focused on SPINs with strong FB connections (Figure 3H, arrows, also see Methods). These SPINs are located in the middle of the atlas, in contrast to LH-, MB-, and OPN-related SPINs on the arms (Figures 1L,N, 3H, Supplementary Figure 6C), indicating integration of broad inputs. To test this, we analyzed SPIN connections with major cell groups across layers (Figure 3I). LHON and MBON neurons are major direct inputs, and FBt neurons are the dominant outputs. Thus, SPINs integrate LHON and MBON inputs and feed into FBt. SPINs send most of the synaptic outputs to FBt (Figures 1H,J, arrows), while receiving a small number of synaptic inputs from FBc, in both hemibrain and FlyWire (Figures 1G,I, 3H). Thus, FB acts as the main output of SPINs, in contrast to LH. To examine the network topology of SPIN-FBt connectivity, we clustered SPINs based on their connections with FB neurons (see Methods). We analyzed the connectivity between SPIN clusters and FBt neurons. Opposite to the many-to-one topology in LHON to SPIN inputs (Figure 3D), SPINs send divergent outputs to many FBt neurons in a one-to-many relation (Figures 3J,K), supporting the convergence-divergence integration model.

### 3.8 MB modulates SPINs via mixed network

The mushroom body (MB) is the center for learned valence and internal state (Davis, 1993; Heisenberg, 2003; Hattori et al., 2017; Cognigni et al., 2018; Cayco-Gajic and Silver, 2019; Sayin et al., 2019; Modi et al., 2020; Zolin et al., 2021). MBONs provide substantial synaptic inputs to SPINs (Figures 1G,I). To examine MB and SPIN interaction, we focused on SPINs with strong MB connections clustered within one arm on the atlas (Figure 3L), indicating a tightly related population. We analyzed these SPIN connections with major cell groups across layers (Figure 3M), and found that both MBONs and LHONs are primary direct inputs. This indicates that MB learned inputs are strongly coupled with LH innate inputs to supply SPIN, in contrast to LH innate inputs that tend to dominate SPIN input (Figure 3B). FBt neurons are the primary outputs, especially via two intermediate layers of SPINs. This again supports that SPINs integrate LHON and MBON inputs into FBt. To examine the topology of MBON-SPIN connectivity, we clustered SPINs based on their connections with MB neurons. We analyzed the connectivity of SPIN clusters with MBONs. Unlike the many-to-one and one-to-one connectivity in LH- and OPN-related SPINs, most MBON types send outputs to multiple SPIN clusters, and most SPIN clusters integrate inputs from multiple MBON types (Figures 3N,O). This forms a many-to-many connectivity, supporting the mixed model.

We found network topologies including many-to-one for LHON-SPIN, one-to-many for SPIN-FBt, many-to-many for MBON-SPIN, and one-to-one for OPN-SPIN, support convergence-divergence integration model, mixed model, and labeled-line model we hypothesized, respectively (Figures 3P,Q). These results illustrate the implementation of diverse networks for information processing at different stages of the SPIN circuit.

### 3.9 Convergence-divergence SPIN network for complex valence processing

Given the convergence-divergence LHON/MBON-SPIN-FBt network topology (Figure 3Q), we wondered what information SPINs might be encoding. Based on our results, we argued that such information should satisfy three conditions: (1), it can be encoded as low-dimensional information in SPINs like in the bottleneck of the autoencoder network; (2), it requires brain-wide multimodal inputs from the convergence network; (3), it needs to be broadcasted via divergence network. Besides, SPINs integrate deep sensory inputs rather than direct ones, suggesting involvement in higher-order functions that require deep sensory inputs. Given that SPIN input LHONs and MBONs are both implicated in valence while output FBt uses valence (Aso et al., 2014b; Das Chakraborty et al., 2022), and valence as a scalar is low dimensional, we hypothesized that SPINs further process valence. We reasoned that valence satisfies the aforementioned conditions. First, low-dimensional constraints exclude complex information such as spatial maps, better represented by population coding, and instead favor simple scalar variables like valence. Second, reliable processing of even a seemingly simple variable of valence is actually quite demanding (Tye, 2018; Tye et al., 2024). Valence information is distributed across modalities (Wang et al., 2018; Wang P. Y. et al., 2020; Das Chakraborty et al., 2022), and thus requires multimodal integration, a feature of SPIN inputs. Related variables such as value, which is also seemingly simple, require complex circuits for its computation (Hu, 2016; Watabe-Uchida et al., 2017; Knudsen and Wallis, 2022) that also exhibit convergence topology (Watabe-Uchida et al., 2017). Third, valence is an important variable that is useful for various functions and needs to be broadcasted. Furthermore, valence processing requires deep sensory information, not sensory features *per se*. We hypothesized that SPINs process valence via a convergence-divergence network, from LHON and MBON inputs to FBt outputs.

To directly address the valence hypothesis, we next focused on specific SPIN circuits, identifying the SMP34x ensemble. Comprising cholinergic and dopaminergic neurons, this ensemble integrates inputs from many LHONs and MBONs and projects to many FBt neurons (Figure 4A, Supplementary Figures 7A–E; see Methods), a pattern consistent between hemibrain and FlyWire (Supplementary Figure 7F). Thus, SMP34x forms a convergence-divergence circuit suitable for multimodal integration and broadcasting of valence signals. To understand the integration process, we focused on the SMP353 neuron, which, similar to other SMP34x neurons, samples broadly from multiple LHONs and MBONs.

Integration of diverse inputs generates increasingly complex representations. In the visual system, for instance, organized integration of excitatory and inhibitory signals from the ON and OFF pathways creates receptive fields with distinct components, enabling feature detection impossible with single components alone (Hubel and Wiesel, 1962; Seelig and Jayaraman, 2013; Sun et al., 2017). This integration can be hierarchically cascaded to form complex cells, facilitating advanced object detection and even visual invariance (Riesenhuber and Poggio, 2000). We asked whether a similar hierarchical, convergent design is employed for complex valence detection. While SMP353 already integrates both LHONs and MBONs, we wondered whether SMP353 receives excitatory or inhibitory inputs alone, or a combination of both. Based on the literature and neurotransmitter predictions, we examined the neurotransmission of SMP353 input cell types. SMP353 integrates cholinergic, GABAergic, and glutamatergic LHON inputs, as well as cholinergic and glutamatergic MBON inputs (Figure 4B). Thus, SMP353 integrates excitatory and inhibitory inputs for innate and learned valences. A related circuit design for reliable valence detection by integrating excitatory and inhibitory inputs has been reported in *C. elegans* (Dobosiewicz et al., 2019), suggesting that this may be a general principle across species. Prior work has shown that SMP353 drives wind-oriented movement (Aso et al., 2023), confirming its role in positive valence. However, the exact function of SMP353 in complex environments remains uncertain. We hypothesized that combining excitatory and inhibitory inputs for innate and learned valences yields complex valence representations, similar to visual receptive fields, making SMP353 SPIN a “complex valence” neuron. To further explore the complex valence hypothesis, we systematically mapped the inputs to SMP353.

### 3.10 SPIN input LHON integrates unimodal and multimodal valences

We began by analyzing LHONs, the predominant input to SMP353. While the LH is known for processing innate valence (Jefferis et al., 2007; Dolan et al., 2018; Das Chakraborty et al., 2022), prior studies largely relied on synthetic monomolecular stimuli. In contrast, natural behaviors like foraging involve complex “olfactory objects” defined by a diverse array of odors (Mansourian and Stensmyr, 2015). We hypothesized that integrating multiple ethologically related odors enhances the robustness of valence signals in natural contexts. Furthermore, while LH has been studied as a whole, the functions of individual LHONs remain largely unknown. To derive these functions at single-cell resolution, we analyzed their synaptic integration rules. While upstream uPNs receive homogeneous inputs from olfactory receptor neuron ORNs expressing the same receptors and generally follow a linear normalization model (Wilson, 2013; Badel et al., 2016; Bates et al., 2020), LHONs and SPINs receive broad multimodal inputs that likely require more complex processing. Indeed, many central brain neurons exhibit nonlinear integration (Tye et al., 2024). This is necessary because single chemicals often possess ambiguous valence depending on the natural olfactory object they belong to, while a single glomerulus can process different chemicals of opposite valences. For example, acetoin and its derivatives elicit opposite valences in different glomeruli (VM3 and VA2), and the single DM3 glomerulus processes both attractive ethyl acetate and aversive pathogenic bacterial product 2,5-dimethylpyrazine (Mathew et al., 2013; Badel et al., 2016; Mohamed et al., 2019). Thus, we hypothesized that LHONs and SPINs integrate inputs nonlinearly. By integrating multiple chemically distinct but ethologically relevant odors, nonlinear integration enables reliable valence detection of natural objects. If this hypothesis is true, we should see the integration of uPN glomeruli with diverse valences that collectively signal a definitive valence of natural object, either attractive or aversive. To test the nonlinear integration hypothesis, we examined LHONs and their upstream uPNs. LHONs consistently integrate diverse inputs from multiple uPNs processing ethologically related odors (Figures 4C–E). For instance, the prominent SMP353 input LHPV6a1 integrates uPN inputs from over twenty glomeruli that process wide-ranging odors such as decaying fruits (DL2d, DL2v), fruity esters (VA3, VM2), and yeast alcoholic fermentation of fruits (DP1l/m, VA2) (de Bruyne et al., 2001; Hallem et al., 2004; Hallem and Carlson, 2006; Silbering et al., 2011; Prieto-Godino et al., 2017; Figure 4C). Although specific glomeruli carry ambiguous valences, such as attractive VA3, VA2, VM2, and aversive DP1m, these glomeruli jointly signal the presence of overripe fruits. This supports nonlinear integration, indicating that LHPV6a1 may mediate attraction by resolving these ambiguous inputs into a coherent food signal. A similar case is LHAD1b2 (Figure 4D). While LHPV6a1 prefers fruit-related odors, LHAD1b2 prefers yeast-related odors, such as yeast alcoholic fermentation (VL2p, DP1l/m, VA2) and yeast volatiles (VA3, DM6) (de Bruyne et al., 2001; Hallem et al., 2004; Hallem and Carlson, 2006; Silbering et al., 2011). Specifically, VL2p, DP1m/l, and VA2 glomeruli process pyruvic acid, 2,3-butanedione, acetoin, and acetic acid, compounds representing distinct steps of the alcoholic fermentation pathway. By integrating across these metabolic steps, LHAD1b2 is positioned to achieve invariant detection of yeast growing on overripe fruits, one of the favorite foods for flies, regardless of its fermentation stage. Integration of attractive (VA3, VA2) and aversive (DP1m) inputs further supports nonlinear integration. Complex natural olfactory objects are composed of multiple related odors instead of single synthetic molecules generally used in the lab. By integrating numerous channels of related odors, the convergence network is well suited for processing complex natural olfactory objects, potentially providing robustness and invariance to valence processing. Such invariant detection of complex objects has also been explored in the visual system using convergence network topology (Riesenhuber and Poggio, 2000). By integrating fruit-preferring LHPV6a1 with yeast-preferring LHAD1b2, alongside other food-related LHONs, SMP353 is well positioned to detect food resources and signals attractive valence for foraging. As an aversive valence example, LHPV4b2 integrates uPN inputs specifically from danger-sensing glomeruli, such as toxic mold and bacteria products like geosmin by DA2 (Stensmyr et al., 2012) and 2-nonanone by DL5 (Mathew et al., 2013; Figure 4E), thus mediating strong aversion.

Natural objects are inherently multisensory. For example, a male fly releases the male-specific volatile pheromone cVA, sings pulse songs to females during courtship, and displays visual cues such as color and motion (Kurtovic et al., 2007; Dickson, 2008; Agrawal et al., 2014; Coen et al., 2014; Clowney et al., 2015; Ribeiro et al., 2018; Deutsch et al., 2019; Baker et al., 2022; Ning et al., 2022; Taisz et al., 2023). To investigate whether the convergence pathway integrates multisensory features, we examined sensory inputs to LHONs. The glutamatergic SMP353 input LHPV4b9 integrates auditory and visual cues. We reasoned that such auditory and visual cues likely signal a social object. Indeed, LHPV4b9 receives input from highly selective pulse song detectors, AVLP_pr36, pC2l, and AVLP_pr12 (Deutsch et al., 2019; Baker et al., 2022), indicating conspecific male detection (Figure 4F). This is complemented by visual inputs from motion detectors T4 and T5, and color vision detectors Tm5 and Tm20 (Song and Lee, 2018; Borst and Groschner, 2023; Christenson et al., 2024; Matsliah et al., 2024). These cues strongly indicate the presence of a conspecific male. We further examined whether olfactory cues reinforce this social signal. LHPV4b9 also integrates olfactory inputs from glomeruli including DA1, VA1d, VA1v, DC3, and DL3 (Figure 4F), all of which respond to pheromones (Ronderos et al., 2014; Dweck et al., 2015; Lin et al., 2016; Sethi et al., 2019). Notably, this includes DA1 and DL3 glomeruli for cVA (Kurtovic et al., 2007; Wang and Anderson, 2010; Liu et al., 2011). Together, these inputs constitute the complete set of major pheromones, providing robust chemosensory detection of conspecifics. By integrating highly specific olfactory, auditory, and visual detectors, LHPV4b9 can achieve specific and invariant conspecific male detection in complex natural environments. Furthermore, LHPV4b9 connects with LHAD1g1, a known multimodal conspecific detector (Taisz et al., 2023), potentially further reinforcing detection robustness. Finally, we observed broad multisensory inputs, indicating that multimodal integration is a general feature of SMP353 inputs (Figure 4G).

### 3.11 SPIN integrates opponent innate valences of attraction and aversion from LHONs

Each LHON input to SMP353 forms a definitive valence signal by multimodal integration. We investigated how SMP353 resolves these opponent valences. We observed a correlation between neurotransmitter identity and valence: attractive LONHs (e.g., LHPV6a1 and LHAD1b series) are cholinergic, whereas aversive LHONs (e.g., LHPV4b2) are glutamatergic (Figures 4B,H). A systematic comparison of upstream uPN inputs confirmed the generality (Figure 4I). Cholinergic LHONs receive substantial inputs from glomeruli associated with yeast and fruit (Semmelhack and Wang, 2009; Mansourian and Stensmyr, 2015), indicative of attractive food cues, while glutamatergic LHONs are dominated by inputs from glomeruli associated with toxic bacteria (Stensmyr et al., 2012), temperature (Gallio et al., 2011; Marin et al., 2020), and humidity (Frank et al., 2017), indicative of aversive environmental cues. Thus, SMP353 combines excitatory attractive inputs with inhibitory aversive inputs. Specifically, SMP353 balances attractive valence of food against aversive valence of toxic bacteria (via glutamatergic LHPV4b2) and conspecific (via glutamatergic LHPV4b9). The glutamatergic nature of LHPV4b9, which encodes social cues, is notable. Actually, pheromone dominates glutamatergic LHON inputs in general, while food-related cues dominate cholinergic LHON inputs (Figure 4I). Since social interaction and foraging are competing goals (Lin et al., 2022), the inhibition of SMP353 by social cues suggests a circuit mechanism for arbitrating between these behaviors, suppressing foraging when potential mates or rivals are present. The integration of opponent valences parallels feature detection in the visual system, where convergence of opponent ON and OFF features generates complex ON-OFF receptive fields (Figure 4J, upper panel). Similarly, the LHON-SPIN convergent integration circuit transforms simple monomeric valence in LHON into a “complex valence receptive field” (Wilson, 2013) in SPIN with both attractive and aversive valence components (Figure 4J, lower panel). This transformation likely enhances performance in natural environments, allowing the organism to map a sophisticated valence landscape rather than simply detecting single cues. In summary, by hierarchical convergence to integrate unimodal and multimodal cues in LHONs, and excitatory attractive and inhibitory aversive valence in SPIN, SMP353 assumes complex multimodal valence receptive fields for robust valence detection. While LH inputs provide a hard-wired innate valence baseline, we asked whether learned valences and internal states also modulate SMP353 for flexible valence processing.

### 3.12 SPIN integrates state-dependent attractive and aversive learned valences from MBONs

To investigate learned valence modulation, we analyzed SMP353 inputs from the mushroom body. The most prominent MB inputs are MBON07 and MBON14 (Figure 4B), both implicated in long-term memory (Plaçais et al., 2013; Aso et al., 2014b). Anatomically, axons of these MBONs, alongside input LHONs, converge with SMP353 dendrites to form a medial-to-lateral corridor spanning SIP and SLP (Aso et al., 2014a,^2023^; Figure 4K), providing a structural basis for valence integration on SMP353 dendrite. Notably, the neurotransmitter logic mirrors that of innate valence: the attractive MBON14 is cholinergic while the aversive MBON07 is glutamatergic. Thus, SMP353 integrates opponent learned valences via the same excitatory/inhibitory mechanism used for innate valence, a design conserved across development (Eschbach et al., 2021). Previous studies have shown innate and learned valence integration via MBON direct modulation of LHON (Dolan et al., 2018). We next examined whether MBONs also indirectly modulate SPIN via LHONs. Monosynaptic connectivity analysis between all MBONs and input LHONs revealed that MBONs preferentially modulate excitatory LHONs, with negligible input to glutamatergic LHONs (Figure 4L, green square). Over ten MBON types, both cholinergic and glutamatergic, strongly modulate cholinergic LHONs most heavily connected with SMP353 (like LHAD1b2, LHAD1b5, and LHPV6a1, Figure 4L). In some LHONs (like LHAD1b5), MBON even outweighs uPN synaptic inputs, facilitating strong modulation of innate valence by learned valence. Internal states, such as hunger, are essential for complex behaviors (Anderson, 2016; Flavell et al., 2022). Hungers signals in MB γ lobe dopaminergic neurons (Cognigni et al., 2018; Lin, 2023) propagate through a layered MB intrinsic network linking γ, β, and α lobes to MBON07 and MBON14, which in turn target SMP353 (Aso et al., 2014b; Li et al., 2020; Figure 4M). Thus, SMP353 is informed by hunger state information via a multilayer circuit originating in the γ lobe. Crucially, MBON07 and MBON14 (Figure 4L, arrows) provide the majority of both direct inputs and indirect inputs to SMP353, constituting a novel two-layer motif where MBONs modulate a feedforward circuit at two levels (Figure 4M). Functionally, this allows bidirectional learned valence and internal states to hierarchically modulate innate food attractions (via excitatory LHONs) for flexible foraging decisions. Conversely, glutamatergic LHONs encoding innate aversion bypass this modulation via hardwired inhibition (Figure 4M), effectively prohibiting foraging decisions in hazardous conditions.

### 3.13 SPIN sends complex valence to FB and MB modulatory neurons via divergence network

SMP353 integrates innate and learned valences via a convergence network of excitatory and inhibitory inputs, creating a hub for complex valence processing. To explore how complex valence is read out, we examined the SMP353 downstream circuit in the fan-shaped body of the central complex. SMP353 heavily innervates tangential neurons in dorsal FB, especially layer 6 (Figures 4A,B). These neurons are implicated in context-dependent action selections related to foraging, metabolism, and sleep, processes critical for homeostasis (Donlea et al., 2011, 2014, 2018; Hu et al., 2018; Dag et al., 2019; Li et al., 2021; Sareen et al., 2021; Lei et al., 2022; Flores-Valle and Seelig, 2023; Goldschmidt et al., 2023). To balance competing needs, like foraging versus energy conservation, flies must weigh internal states against external risks and opportunities. By integrating multimodal inputs from LHONs and MBONs, SPIN-FB pathway provides a circuit basis for context-dependent action selection. Given the high level of processing, SPINs should broadcast outputs broadly via the divergence network. Indeed, SPINs project not only to FB but also to MB DANs, and diverse modulatory systems (dopaminergic, serotonergic, octopaminergic, and peptidergic neurons, Figure 4A). This divergence network provides a well-suited structural basis that allows SPIN valence signals to influence global brain function.

### 3.14 SPIN dendritic integration of conflicting valences

Single SMP353 neuron integrates complex, potentially conflicting synaptic inputs. To understand how a single cell resolves conflicts between attractive/aversive and innate/learned signals, we reasoned that evolutionarily optimized priority rules might shape its subcellular structure. We hypothesized that in attractive valence neurons like SMP353, innate aversion should be prioritized over broad attraction, while learned valence should be prioritized over innate valence for behavioral flexibility. Such priority rules can be implemented via dendritic computation. To test the hypotheses, we examined synaptic organization of SMP353. To facilitate topological analysis, we mapped 3D synaptic locations onto a 2D dendrogram for SMP353 (Figures 5A,B, also see Methods). Neurons often assume a compartmentalized structure (Kondo et al., 2020; Liu et al., 2022; Sanfilippo et al., 2024), where inputs and outputs are separated by the site of action potential initiation (Figures 5A,B). Although many fly neurons exhibit intermixed processes, our analysis of SMP353 revealed a clear spatial segregation (Figure 5C). Pre-synaptic sites onto FB neurons are clustered together on the opposite side of the putative action potential initiation site than post-synaptic sites (Figures 5B,C), assuming that the branch point of the axonal processes from the primary process designates the site of action potential initiation (Gouwens and Wilson, 2009), indicating compliance with compartmentalization model.

For survival, aversive valences, like toxic bacteria, must be prioritized over attractive valences, like yeast food. To investigate how SMP353 solves this conflict via dendritic computation, we examined the organization of its synaptic inputs. Inhibitory synapses are known to block excitatory inputs via shunting inhibition, mediated by chloride-permeable ionotropic GABA receptors in mammals (Burman et al., 2023), and chloride-permeable ionotropic glutamate receptor GluClα in *Drosophila* (Liu and Wilson, 2013; Groschner et al., 2022; Ammer et al., 2023). Given that SMP353 integrates synaptic inputs for both attractive (excitatory) and aversive (inhibitory) innate valence, we hypothesized that inhibitory aversive inputs are positioned closer to the action potential initiation site to block excitatory attractive inputs. Mapping the locations of cholinergic and glutamatergic synapses onto the dendrogram (Figures 5D,E) confirmed that glutamatergic synapses are indeed located more proximally than cholinergic synapses (Figures 5D–F). Asking whether synapses for shunting inhibition are clustered to form a potent centralized inhibition or distributed on the dendritic tree to form hierarchically inhibition, we found that glutamatergic synapses tend to be located on dendritic trunks near branches at various stages of the dendritic tree, forming hierarchical shunting inhibition (Figures 5E,F, green arrows). Intriguingly, glutamatergic synapses are significantly fewer in number than cholinergic ones (Figures 5D,E). For such sparse inhibition to be effective, individual glutamatergic synapses should be significantly stronger than cholinergic ones, consistent with reports that GluClα receptors can be tenfold stronger than cholinergic ones (Ammer et al., 2023). Structural analysis revealed the basis for this strength: (Figures 5G–L, Supplementary Figure 8) a spine-like enlarged protrusion on the SMP353 dendrite (Figures 5K,L) apposed to a bouton from LHPV4b2 primary neurite (Figures 5J,K), forming a prominent “root” synapse (Figures 5G–L, Supplementary Figure 8) strategically positioned to gate upstream cholinergic inputs via potent shunting inhibition.

To adapt to changing circumstances, learned valences must override innate valences to drive behavior. For example, an odor that innately signals food might become undesirable if that food recently led to sickness. As SMP353 integrates both innate (LHON) and learned (MBON) valences, we examined how it solves the conflict via dendritic computation. We hypothesized that MBON synapses should be positioned more proximally than LHON ones to override innate valence when circumstances change. Consistent with this, the dominant LHON inputs are excitatory, while the dominant MBON inputs are inhibitory (Figure 4B). This arrangement supports a shunting inhibition mechanism, where inhibitory MBON learned valence effectively override excitatory LHON innate valence. Indeed, MBON synapses are located more proximally than LHON synapses (Figures 5M–S). Specifically, a glutamatergic MBONα1 synapse is located on the SMP353 dendritic trunk (Figures 5N,O), effectively gating this dendrite. Across the SMP353 dendritic tree, MBON proximal arrangement provides a structural basis for MBONs to modulate LHON inputs (Figures 5P–S).

Together, we found a hierarchical dendritic organization, where glutamatergic MBON synapses gate LHON inputs, and glutamatergic LHON synapses gate cholinergic ones. By delicate dendritic organization of conflicting synaptic inputs, SPINs prioritize ethologically critical valence signals.

### 3.15 Glutamatergic SIP015 integrates multimodal inputs into complex aversive valence

SMP34x ensemble exemplifies a convergence-divergence network from multimodal inputs to FB outputs for valence processing via dendritic computation. SIP01x ensemble exhibits similar patterns (Figure 6A, Supplementary Figure 9), integrating broad LHON inputs and projecting divergently to FBt in both hemibrain and FlyWire (Supplementary Figure 9D). Unlike the single cholinergic SMP353, SIP015 within SIP01x comprises 4 (hemibrain) to 6 (FlyWire) glutamatergic neurons per hemisphere (Supplementary Figures 9A–C). SIP015 integrates a wide array of LHON and MBON excitatory and inhibitory inputs (Figure 6B), akin to SMP353. Unlike SMP353, where excitatory LHON and inhibitory MBON inputs dominate, we found a higher proportion of inhibitory LHON and excitatory MBON inputs in SIP015. Together with its glutamatergic identity, these connectivity patterns suggest that SIP015 may mediate a function opposite to SMP353, potentially encoding aversive valence.

Ethologically relevant sensory inputs converge onto SIP015, paralleling SMP353. Glutamatergic LHPD2d1 relays mechanosensory signals monosynaptically from WEDPN3 (Bates et al., 2020) and WEDPN12 (also called WED_pr01 and WPNb) (Suver et al., 2019; Coates et al., 2020; Baker et al., 2022), and disynaptically from WEDPN6A and WEDPN8C via interneurons like WEDPN3 (Figure 6C). These WEDPNs process acoustic white noise and wind direction (Suver et al., 2019; Bates et al., 2020; Baker et al., 2022), with morphologically similar WPNs linearly encoding wind direction from broad wedge inputs (Suver et al., 2019). Comprising inhibitory (GABAergic WEDPN3/6A, glutamatergic WEDPN12) and excitatory (cholinergic WEDPN8C) neurons, with an inhibition bias, these WEDPNs bidirectionally signal aversive-biased wind cues. LHPD2d1 thus unimodally integrates mechanosensory cues to convey wind signals to SIP015.

Multimodal integration yields complex valence, paralleling SMP353. Glutamatergic LHPD2d2 fuses thermosensory/hygrosensory inputs: cold (GABAergic VP3), warm/humid (cholinergic VP1m), hot (cholinergic VP2) (Figures 6C,E), plus circadian DN1a (Alpert et al., 2020; Li et al., 2024; Reinhard et al., 2024), signaling environmental conditions spanning aversive coldness to attractive warm and humid conditions for flies (Marin et al., 2020). SIP015 also integrates olfactory inputs, aversive VA6/DC1 glomeruli via LHPD2d2 (Schlief and Wilson, 2007; Mathew et al., 2013; Badel et al., 2016); V glomeruli for CO_2_ via LHPD2d1/LHPD2d2 (Figure 6E, yellow square). CO_2_ from various sources is generally aversive to flies, with state-dependent complexity (Suh et al., 2004; Faucher et al., 2006; Jones et al., 2007, Ai et al., 2010; Lin et al., 2013; van Breugel et al., 2018), underscoring the importance of putting features into context. SIP015 thus integrates multisensory LHON inputs, both excitatory attractive and inhibitory aversive (Figure 6F), both chemosensation and physical sensation (Figures 6D,E), to signal innate aversion. Combining wind, temperature, humidity, circadian, and olfactory cues, SIP015 reliably detects conditions unfit for exploration. Thus, while SMP353 depicts an attractive chemosensory scene rich in food, SIP015 portrays an aversive physical condition characterized by inclement weather.

Like SMP353, SIP015 integrates innate and learned valences from LHONs and MBONs (Figure 6B), with a higher MBON proportion, including both cholinergic and glutamatergic MBONs. Cholinergic MBONs comprise vertical lobe V3/4 and V2 clusters for long-term memory retrieval (Séjourné et al., 2011; Plaçais et al., 2013; Aso et al., 2014b; Bouzaiane et al., 2015): MBON13 (α’2), MBON12 (γ2α’1), MBON14 (α3), MBON17 (α’3m), MBON18 (α2sc), and MBON19 (α2p3p). Glutamatergic MBONs include comprising horizontal lobe M4/6 cluster: MBON1 (γ5β’2a), MBON2 (β2β’2a), and MBON3 (β’2mp). These cholinergic and glutamatergic MBONs signal positive and negative valence, respectively. Thus, SIP015 integrates excitatory attractive and inhibitory aversive MBONs, similar to SMP353, indicating a general design principle. Like two-layer MBON modulation in SMP353, prominent SIP015 input MBONs also target its input LHONs (Figure 6G). Cholinergic (MBON18/13/12) and glutamatergic (MBON04/03/01) MBONs target cholinergic LHPV5e1/LHPV10d1. Specifically, MBON18 (MBONα2sc), implicated in memory retrieval and interaction with LHONs (Séjourné et al., 2011; Hige et al., 2015; Dolan et al., 2018), provides strong inputs to LHPV5e1/LHPV10d1, indicating similar MB-LB interaction functions. Unlike SMP353 where MBONs exclusively target cholinergic LHONs, MBONs target both cholinergic and glutamatergic input LHONs in SIP015 (Figure 6G). For example, cholinergic (MBON23/13/12) and glutamatergic (MBON02/03/04) MBONs target glutamatergic LHPD2d1. All three MBON input pathways-monosynaptic, disynaptic via cholinergic LHONs, and disynaptic via glutamatergic LHONs-arise from identical MB compartments: cholinergic α2, α’2, and α’1 (MBON23/18/13/12), and glutamatergic β’2 (MBON02/03/04), a pattern similar to SMP353 except for the dominant glutamatergic LHON pathway (Figure 4M), suggesting a more complex version of the two-layer MBON modulation circuit model.

SIP015 targets multiple FB tangential neurons (Figure 6B), akin to SMP353. Together, we discovered that SIP015 integrates multimodal innate and learned inputs into FB via a convergence and divergence network, similar to SMP353 but differ in LHON/MBON composition: cholinergic SMP353 contains more cholinergic LHON and glutamatergic MBON, glutamatergic SIP015 contains more glutamatergic LHON and cholinergic MBON, exhibiting symmetric opponency.

SMP353-SIP015 symmetry extends to dendritic implementation. SIP015 pre-synaptic sites onto FB tangential neurons cluster near the putative action potential initiation site, indicating compartmentalization (Figures 6H–J). Cholinergic LHON synapses distribute downstream of glutamatergic LHON inputs (Figures 6K–M, pink arrows), opposite to the pattern in SMP353. Similarly, LHON synapses tend to lie downstream of MBON synapses (Figures 6N–S). Thus, aversive SIP015 prioritize innate aversion over learned ones, while innate attraction over aversion, opposite SMP353. This synaptic distribution pattern correlates with synapse strengthens, as SIP015 exhibits higher MBON-LHON and glutamatergic-cholinergic synapse proportions than SMP353. SIP015 dendritic integration patterns thereby symmetrically oppose SMP353, underpinning valence opponency.

## 4 Discussion

Transforming complex sensory information into valence is vital for complex behaviors in natural environments. Significant progress has been made in valence processing with simple synthetic stimuli (Semmelhack and Wang, 2009; Choi et al., 2011; Badel et al., 2016), yet mechanisms for complex valence processing for natural objects remain unclear. We identified a convergence-divergence SPIN circuit that integrates multimodal innate and learned opponent valences from LH and MB, and discovered circuit and dendritic organizations underpinning complex valence processing in *Drosophila*, in both hemibrain and FlyWire connectome datasets. These structural organization rules may represent generalizable principles for complex valence processing.

### 4.1 Convergence-divergence network for complex valence processing

**FIGURE 7:**
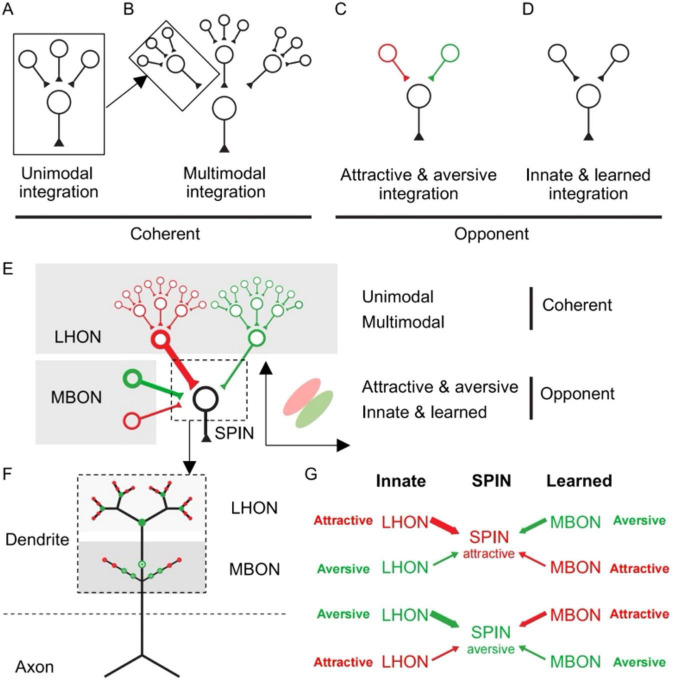
Hierarchical SPIN motif, circuit, and dendritic models for complex valence integration. **(A–D)** Schematic of four types of convergent circuits for coherent **(A,B)** and opponent **(C,D)** integration of valences discovered in this study. Coherent integration includes both unimodal **(A)** and multimodal **(B)** integration, where a hierarchical combination of unimodal integration circuits gives rise to a multimodal integration circuit. Opponent integration includes the integration of attractive (red) and aversive (green) valences **(C)** and the integration of innate and learned valences **(D)**. Integrating excitatory attractive and inhibitory aversive valences generally applies to both innate and learned valences. These four circuit motifs constitute building blocks for more complex valence integration circuits. **(E)** Schematic of hierarchical convergent integration SPIN input circuit, by the combinatorial use of the four types of circuit motifs **(A–D)**. **(F)** Schematic for dendritic integration of opponent valences. Glutamatergic synapses tend to locate more proximally than cholinergic synapses, providing a single cell structure for complex valence integration via shunting inhibition, both for aversive overriding attractive valences and for learned overriding innate valences. **(G)** Symmetrically opponent organization in convergence integration circuits. Cholinergic attractive SPIN, like SMP353, receives more cholinergic than glutamatergic LHON inputs, but more glutamatergic than cholinergic MBON inputs; while glutamatergic aversive SPIN, like SIP015, exhibits an opposite organization.

Along the valence transformation, such as the uPN-LHON/MBON-SPIN pathway we studied, the AL, the LH, and the MB have been implicated in valence processing. Here, we extended this valence pathway into SP. Why are there multiple layers of valence processing? We reason that as valence is critical for survival, the brain must consider all possible factors to ensure its reliability and robustness. Although valence signals appear simple and low-dimensional, deducing high-dimensional multimodal cues and experience from the natural environment into simple valence signals for actions is demanding. By flexible use of the convergence-divergence motif, the SPIN circuit evolved four solutions to this challenge: integrating relevant cues within modality, integrating multiple cues across modalities, combining attractive and aversive opponent valences, and updating innate valence with learned valence (Figures 7A–D). These four solutions are jointly applied to individual SPIN neuron input circuits (Figure 7E). Besides, unimodal and multimodal convergent integration of coherent valences generates monomeric valence in LHONs, while convergent integration of attractive-aversive and innate-learned opponent valences gives rise to complex valence in SPINs. This monomeric to complex valence transformation defines the functions of LHONs and SPINs as well as the convergence circuit. During interactions with natural objects such as food or mates, animals must parse complex sensory cues from the object and robustly transform them into valence to select appropriate actions. However, valence processing is traditionally studied using simple stimuli, such as synthetic chemicals (Semmelhack and Wang, 2009; Choi et al., 2011; Fosque et al., 2015; Badel et al., 2016). Natural objects tend to exhibit complex yet related sensory profiles that reflect complementary aspects of the object under scrutiny, such as a group of associated odors (Mansourian and Stensmyr, 2015) or different views of an object (Logothetis et al., 1994; Ning et al., 2022). Combining related cues improves the invariance of identification and robustness of valence detection under complex and dynamic environments. Related cues include both single-modality cues, such as associated odors of a specific food, and multi-modality cues, such as male pheromones and courtship songs. SPIN input LHON convergence circuits integrate both single modality cues, such as the complete set of pheromones, and key steps of yeast alcoholic fermentation pathway, and multi-modality cues such as olfactory, auditory, and visual tri-modality integration of conspecific male cues (Figures 7A,B).

In more challenging cases, valence signals can be ambiguous or even conflicting, such as a food source with weak signatures of bacterial infection. Animals must evaluate conflicting evidence of both edibility and harmfulness to balance cost and benefit. In stark contrast to uPNs and LHONs, which generally exhibit monomeric valence of either attraction or aversion, SPINs integrate attractive and aversive valences (Figure 7C). Such valence opponency design is similar to visual receptive fields combining ON and OFF pathways via excitation and inhibition for complex feature detection (Seelig and Jayaraman, 2013; Sun et al., 2017), as well as motion and color opponency (Mauss et al., 2015; Schnaitmann et al., 2018; Ammer et al., 2023). Furthermore, to facilitate consistent valence computation, the convergence network is organized to mediate attractive and aversive valences by excitatory and inhibitory synapses, respectively. We can further reason that by flipping the sign on the convergence motif such that attractive and aversive valences are integrated with the same sign of transmission, either excitatory or inhibitory, SPIN neurons can compute salience based on the same set of valence inputs. Indeed, valence and salience are processed in the same region, including the mushroom body and the amygdala (Aso et al., 2014b; Gore et al., 2015; Hattori et al., 2017; Yang et al., 2023).

In even more challenging scenarios, typically attractive or aversive cues may switch their valence based on recent experiences. To flexibly update valence in complex behaviors, innate valence must be integrated with learned valence (Figure 7D). Valence processing is segregated from sensory inputs into innate and learned pathways across species (Choi et al., 2011; Fulton et al., 2024), and identifying the hub of innate and learned valence pathway integration is a critical question across model organisms (Gore et al., 2015; Isosaka et al., 2015). Recent studies have examined learned and innate valence interaction in LH and MB (Dolan et al., 2018; Huang et al., 2024). However, whether the key integration process takes place within LH or MB, or in a separate hub is unclear. We identified the SP region that deeply integrates valence from both LH and MB (Figure 7E). Convergence neurons (CNs) that integrate innate and learned valence have been reported in the fly larva brain, which also integrate attractive and aversive learned valences from vertical and horizontal lobe MBONs via excitatory and inhibitory pathways (Eschbach et al., 2021), similar to SPINs. It is intriguing whether larval CNs and adult SPINs are developmentally related. Another factor where subjective experience affects valence processing is the internal state, such as the hunger state biasing the choice of contaminated food. We found SPIN modulation by MB and other modulatory neurons that have been implicated in internal state representation. By combining learning with the internal state via the convergence network, SPINs can update valence flexibly and timely.

While the neural circuits for sensory feature detection have been heavily studied (Harris and Mrsic-Flogel, 2013), those for valence processing are less understood. Although circuit motifs have been proposed for valence-driven behavioral outputs (Tye, 2018), neural circuits transforming sensory inputs into complex valence signals remain unclear. Oppositely structured to the divergence-convergence network in the PN-KC-MBON circuit implicated in pattern separation (Cayco-Gajic and Silver, 2019), we identified the convergence-divergence network of the LHON/MBON-SPIN-FBt circuit. Interestingly, the convergence-divergence network is essentially an autoencoder network, widely used for representation learning and generative models (Lecun and Soulie Fogelman, 1987; Kramer, 1991; Goodfellow et al., 2014). Autoencoder network features low-dimensional latent representations that extract key information while suppressing noise from convergent inputs to reconstruct the inputs as divergent outputs. Given that SP receives inputs from nearly the entire brain, SPINs are uniquely positioned to compute valence in complex natural environments and broadcast this information to inform goal-directed action planning in FB.

### 4.2 Dendritic integration of valence opponency

We identified an excitation-inhibition convergence network mediating multimodal integration of appetitive and aversive, as well as innate and learned valence opponency (Figure 7E). Interestingly, we found symmetric but opponent structural organization in the SMP353 and SIP015 convergent input networks, reflecting their valence opponency (Figure 7G). Strikingly, such a convergence network, which samples information from across most of the brain, converges onto a single SPIN neuron (Figure 7F). Why such a design? What function would single neuron implementation confer that is harder with neuron populations? We reasoned that such opponency integration is required for complex environments with opponent valences, where the coincidence of both valences activates the SPIN, potentially for multiplication to signal the spatiotemporal contingency. Such multiplication can be implemented with multiplicative disinhibition, through inhibitory glutamatergic transmission via the GluClα channel (Groschner et al., 2022; Ammer et al., 2023). In line with this, we found the SPIN aversive inputs to be glutamatergic (Figure 7G). Similar single-cell computations have been reported for visual motion and color opponency, as well as for visual feature and auditory coincidence detection, among others (Peña and Konishi, 2001; Gabbiani et al., 2002; Lin et al., 2014; Klapoetke et al., 2017; Groschner et al., 2018; Schnaitmann et al., 2018; Amin et al., 2020; Ammer et al., 2023). Interestingly, the glutamatergic shunting inhibition in SPINs is provided by bacteria infection, conspecific males, and learned aversion, indicating the ethological priority of these valences.

## Data Availability

The original contributions presented in this study are included in the article/Supplementary material, further inquiries can be directed to the corresponding author.

## Footnotes

1
https://github.com/lmcinnes/umap

2
https://github.com/navis-org/navis

3
https://github.com/aplbrain/dotmotif

4
https://github.com/google/neuroglancer

5
https://molview.org
