# Supplementary material for: Convergence-divergence circuits for multimodal integration of innate and learned opponent valences
Source: Front Syst Neurosci. 2026 May 7;20:1822122. doi: 10.3389/fnsys.2026.1822122 (PMC13190573; doi:10.3389/fnsys.2026.1822122)
Supplement: Supplementary file 4 [file Data_Sheet_1.pdf]

Supplementary Material

1 Supplementary Figures

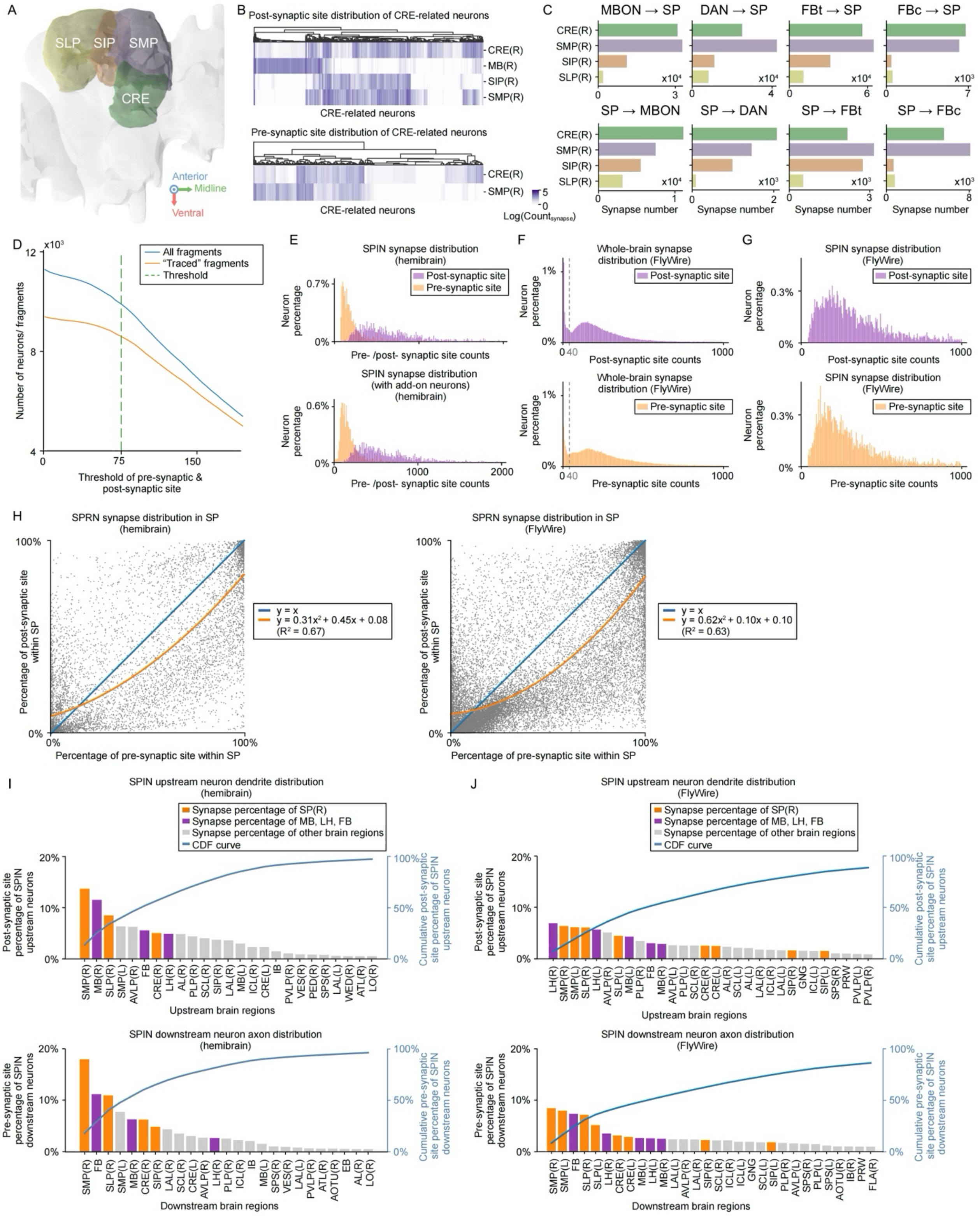
A
SLP
SIP
SMP
CRE
Anterior
Midline
Ventral
B
Post-synaptic site distribution of CRE-related neurons
CRE(R)
MB(R)
SIP(R)
SMP(R)
CRE-related neurons
Pre-synaptic site distribution of CRE-related neurons
CRE(R)
SMP(R)
CRE-related neurons
5
0
Log(Count_synapse)
C
MBON → SP
DAN → SP
FBt → SP
FBc → SP
SP → MBON
SP → DAN
SP → FBt
SP → FBc
CRE(R)
SMP(R)
SIP(R)
SLP(R)
x10⁴
x10³
Synapse number
D
x10³
All fragments
"Traced" fragments
Threshold
Number of neurons/ fragments
Threshold of pre-synaptic & post-synaptic site
E
SPIN synapse distribution (hemibrain)
Post-synaptic site
Pre-synaptic site
Neuron percentage
Pre- /post- synaptic site counts
SPIN synapse distribution (with add-on neurons) (hemibrain)
F
Whole-brain synapse distribution (FlyWire)
Post-synaptic site
Post-synaptic site counts
Pre-synaptic site
Pre-synaptic site counts
G
SPIN synapse distribution (FlyWire)
Post-synaptic site
Post-synaptic site counts
Pre-synaptic site
Pre-synaptic site counts
H
SPRN synapse distribution in SP (hemibrain)
y = x
y = 0.31x² + 0.45x + 0.08
(R² = 0.67)
SPRN synapse distribution in SP (FlyWire)
y = x
y = 0.62x² + 0.10x + 0.10
(R² = 0.63)
Percentage of post-synaptic site within SP
Percentage of pre-synaptic site within SP
I
SPIN upstream neuron dendrite distribution (hemibrain)
Synapse percentage of SP(R)
Synapse percentage of MB, LH, FB
Synapse percentage of other brain regions
CDF curve
Post-synaptic site percentage of SPIN upstream neurons
Cumulative post-synaptic site percentage of SPIN upstream neurons
Upstream brain regions
SPIN downstream neuron axon distribution (hemibrain)
Pre-synaptic site percentage of SPIN downstream neurons
Cumulative pre-synaptic site percentage of SPIN downstream neurons
Downstream brain regions
J
SPIN upstream neuron dendrite distribution (FlyWire)
Synapse percentage of SP(R)
Synapse percentage of MB, LH, FB
Synapse percentage of other brain regions
CDF curve
Upstream brain regions
SPIN downstream neuron axon distribution (FlyWire)
Downstream brain regions

Figure S1. Definition of SP and SPIN. (A) SP in the right hemisphere of the *Drosophila* brain in anterior view, including SLP (yellow), SIP (orange), SMP (purple), and CRE (green). **(B)** Logarithmetic post-/pre- synaptic count distribution (upper/lower panel) of CRE-related neurons. Each column denotes a single neuron. Each row indicates a brain region connected with more than half of the CRE-related neurons. **(C)** Connectivity strengths of SMP, SIP, SLP, and CRE as downstream (upper panels) and upstream (lower panels) of MBON, DAN, FBt, and FBc, shown in total synapse counts. **(D)** Distribution of total synapse count of well-traced (orange) and all fragments (blue) of SP-related neurons in hemibrain. Based on the inflection points of the two curves, we set 75 synapses as the threshold for defining SP-related neurons. See Methods for details. **(E)** Distribution of SPIN pre- and post-synaptic sites in hemibrain. The upper panel shows the synapse distribution of SPINs defined by synapse threshold in **(D)** (over 75 pre- and post-synaptic sites), where the distribution of pre-synaptic sites seems truncated on the left edge, indicating improper classification. The lower panel shows the synapse distribution of SPIN after expansion in cell number while maintaining the number of cell types to capture the complete class, which included all smaller fragments (less than 75 pre- and post-synaptic sites) belonging to the same 585 SPIN types shown in the upper panel. See Methods for details. **(F)** Distribution of total post- (upper panel) and pre- (lower panel) synaptic site count of all neurons across the brain in FlyWire. Dashes denote the threshold for filtering incomplete neurons. **(G)** Distribution of post- (upper panel) and pre- (lower panel) synaptic site count of all SPINs in FlyWire. **(H)** SP-related neuron synapse location distribution in SP without binning in hemibrain (left) and FlyWire (right). The horizontal axes show the percentage of pre-synaptic sites, while the vertical axes show the percentage of post-synaptic sites, with each point denoting a single SP-related neuron. Blue curve, $y = x$; orange curve, polynomial fitting curve of SP-related neuron post-synaptic site versus pre-synaptic site distributions in SP, hemibrain (left), $y = 0.31x^2 + 0.45x + 0.08$, $R^2 = 0.67$; FlyWire (right), $y = 0.62x^2 + 0.10x + 0.10$, $R^2 = 0.63$. **(I-J)** SPIN connectivity preferences to different brain regions in terms of post-synaptic site distribution of upstream neurons (upper panel) and pre-synaptic site distribution of downstream neurons (lower panel) in hemibrain **(I)** and FlyWire **(J)**. Orange and purple colors indicate SP and SP-related neuropils, respectively, as in Figure 1A-B. The blue curves are the cumulative distribution. See Figure 1-table 1 for abbreviations.

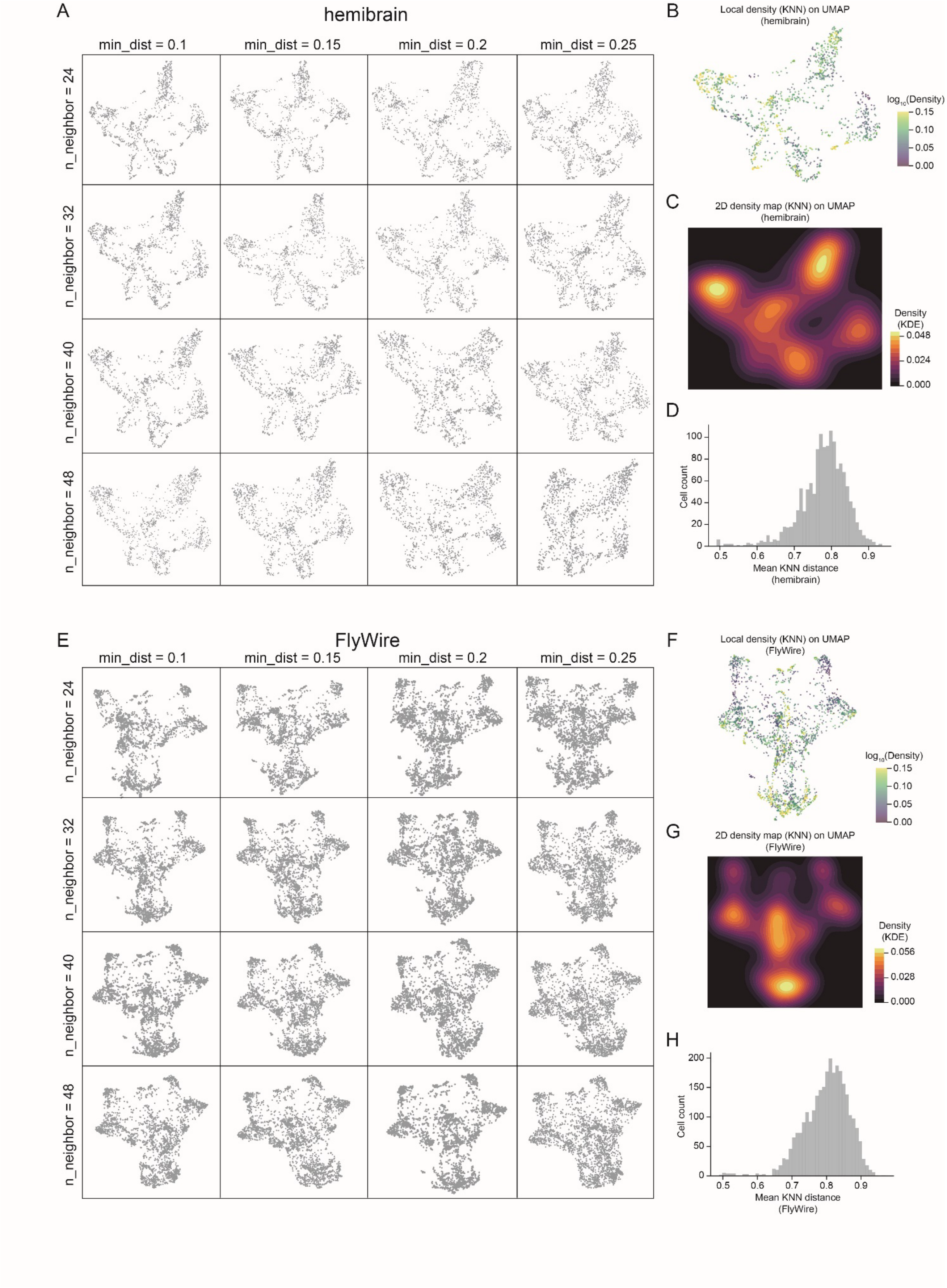
A
hemibrain
min_dist = 0.1
min_dist = 0.15
min_dist = 0.2
min_dist = 0.25
n_neighbor = 24
n_neighbor = 32
n_neighbor = 40
n_neighbor = 48
B
Local density (KNN) on UMAP
(hemibrain)
log10(Density)
0.15
0.10
0.05
0.00
C
2D density map (KNN) on UMAP
(hemibrain)
Density
(KDE)
0.048
0.024
0.000
D
Cell count
100
80
60
40
20
0
0.5
0.6
0.7
0.8
0.9
Mean KNN distance
(hemibrain)
E
FlyWire
min_dist = 0.1
min_dist = 0.15
min_dist = 0.2
min_dist = 0.25
n_neighbor = 24
n_neighbor = 32
n_neighbor = 40
n_neighbor = 48
F
Local density (KNN) on UMAP
(FlyWire)
log10(Density)
0.15
0.10
0.05
0.00
G
2D density map (KNN) on UMAP
(FlyWire)
Density
(KDE)
0.056
0.028
0.000
H
Cell count
200
150
100
50
0
0.5
0.6
0.7
0.8
0.9
Mean KNN distance
(FlyWire)

Figure S2. Robustness and density landscape of SPIN whole-brain connectivity UMAP embeddings in hemibrain and FlyWire. (A, E) UMAP embeddings of SPIN whole-brain connectivity in hemibrain **(A)** and FlyWire **(E)**, generated by systematically varying n_neighbors (24, 32, 40, 48) and min_dist (0.1, 0.15, 0.2, 0.25). Each dot denotes a single SPIN. **(B, F)** Local density of each SPIN computed in the original connectivity space (k=15 nearest neighbors, Bray-Curtis distance) and projected onto the UMAP embedding of hemibrain **(B)** and FlyWire **(F)**. Color denotes base-10 logarithm of local density. **(C, G)** Two-dimensional kernel density estimate (KDE) on the hemibrain **(C)** and FlyWire **(G)** UMAP embedding, showing the density landscape of the SPIN population. Brighter regions indicate higher similarity in connectivity profiles. **(D, H)** Distribution of the mean k-nearest-neighbor (kNN) distances (Bray-Curtis) across all SPINs in hemibrain **(D)** and FlyWire **(H)**.

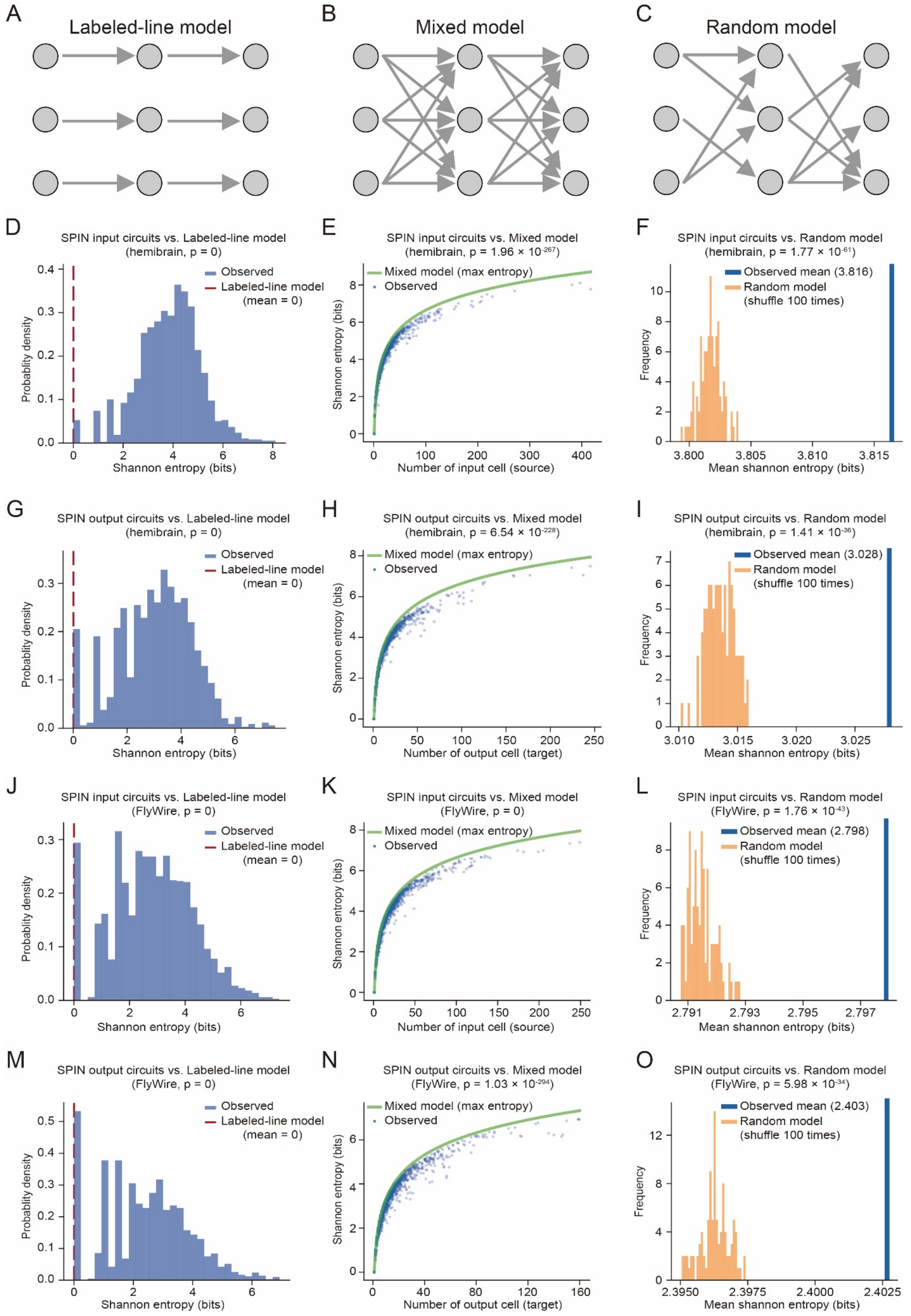
A
Labeled-line model
B
Mixed model
C
Random model
D
SPIN input circuits vs. Labeled-line model
(hemibrain, p = 0)
Observed
Labeled-line model
(mean = 0)
Probability density
Shannon entropy (bits)
E
SPIN input circuits vs. Mixed model
(hemibrain, p = 1.96 × 10-267)
Mixed model (max entropy)
Observed
Shannon entropy (bits)
Number of input cell (source)
F
SPIN input circuits vs. Random model
(hemibrain, p = 1.77 × 10-61)
Observed mean (3.816)
Random model
(shuffle 100 times)
Frequency
Mean shannon entropy (bits)
G
SPIN output circuits vs. Labeled-line model
(hemibrain, p = 0)
Observed
Labeled-line model
(mean = 0)
Probability density
Shannon entropy (bits)
H
SPIN output circuits vs. Mixed model
(hemibrain, p = 6.54 × 10-228)
Mixed model (max entropy)
Observed
Shannon entropy (bits)
Number of output cell (target)
I
SPIN output circuits vs. Random model
(hemibrain, p = 1.41 × 10-36)
Observed mean (3.028)
Random model
(shuffle 100 times)
Frequency
Mean shannon entropy (bits)
J
SPIN input circuits vs. Labeled-line model
(FlyWire, p = 0)
Observed
Labeled-line model
(mean = 0)
Probability density
Shannon entropy (bits)
K
SPIN input circuits vs. Mixed model
(FlyWire, p = 0)
Mixed model (max entropy)
Observed
Shannon entropy (bits)
Number of input cell (source)
L
SPIN input circuits vs. Random model
(FlyWire, p = 1.76 × 10-43)
Observed mean (2.798)
Random model
(shuffle 100 times)
Frequency
Mean shannon entropy (bits)
M
SPIN output circuits vs. Labeled-line model
(FlyWire, p = 0)
Observed
Labeled-line model
(mean = 0)
Probability density
Shannon entropy (bits)
N
SPIN output circuits vs. Mixed model
(FlyWire, p = 1.03 × 10-294)
Mixed model (max entropy)
Observed
Shannon entropy (bits)
Number of output cell (target)
O
SPIN output circuits vs. Random model
(FlyWire, p = 5.98 × 10-34)
Observed mean (2.403)
Random model
(shuffle 100 times)
Frequency
Mean shannon entropy (bits)

Figure S3. Shannon entropy analysis of SPIN input and output circuits. (A-C) Schematics of the labeled-line **(A)**, mixed **(B),** and random **(C)** models. **(D, G)** Shannon entropy distributions for SPIN input **(D)** and output **(G)** circuits in hemibrain. Dashed lines (dark red) indicate the mean entropy of the labeled-line null. The observed entropy is significantly higher than the labeled-line null (input, $p = 0$; output, $p = 0$; one-sample t-test), ruling out a strict labeled-line model. **(E, H)** Shannon entropy plotted against the number of input sources **(E)** or output targets **(H)** for each SPIN (blue dots) in hemibrain. The green curves represent the theoretical maximum entropy for a mixed model distribution. We observed that 97.8% of input and 94.0% of output SPINs fall below this maximum. The entropy deficit is significantly less than zero (input: $p = 1.96 \times 10^{-267}$; output: $p = 6.54 \times 10^{-228}$; one-sample t-test), ruling out the mixed model. **(F, I)** Observed mean entropy (blue line) compared to the distribution of mean entropy from 100 shuffled random networks (orange histogram) for SPIN input **(F)** and output **(I)** circuits in hemibrain. The observed mean entropy deviates significantly from the random null model (input, $p = 1.77 \times 10^{-61}$; output, $p = 1.41 \times 10^{-36}$, z-test), confirming that specific wiring pattern determines connectivity diversity. **(J, M)** Shannon entropy distributions for SPIN input **(J)** and output **(M)** circuits in FlyWire. Dashed lines (dark red) indicate the mean entropy of the labeled-line null. The observed entropy is significantly higher than the labeled-line null (input, $p = 0$; output, $p = 0$; one-sample t-test), ruling out a strict labeled-line model. **(K, N)** Shannon entropy plotted against the number of input sources **(K)** or output targets **(N)** for each SPIN (blue dots) in FlyWire. The green curves represent the theoretical maximum entropy for a mixed model distribution. We observed that 92.8% of input and 87.7% of output SPINs fall below this maximum. The entropy deficit is significantly less than zero (input: $p =0$; output: $p = 1.03 \times 10^{-294}$; one-sample t-test), ruling out the mixed model. **(L, O)** Observed mean entropy (blue line) compared to the distribution of mean entropy from 100 shuffled random networks (orange histogram) for SPIN input **(L)** and output **(O)** circuits in FlyWire. The observed mean entropy deviates significantly from the random null (input, $p = 1.76 \times 10^{-43}$; output, $p = 5.98 \times 10^{-34}$, z-test), confirming that specific wiring pattern determines connectivity diversity.

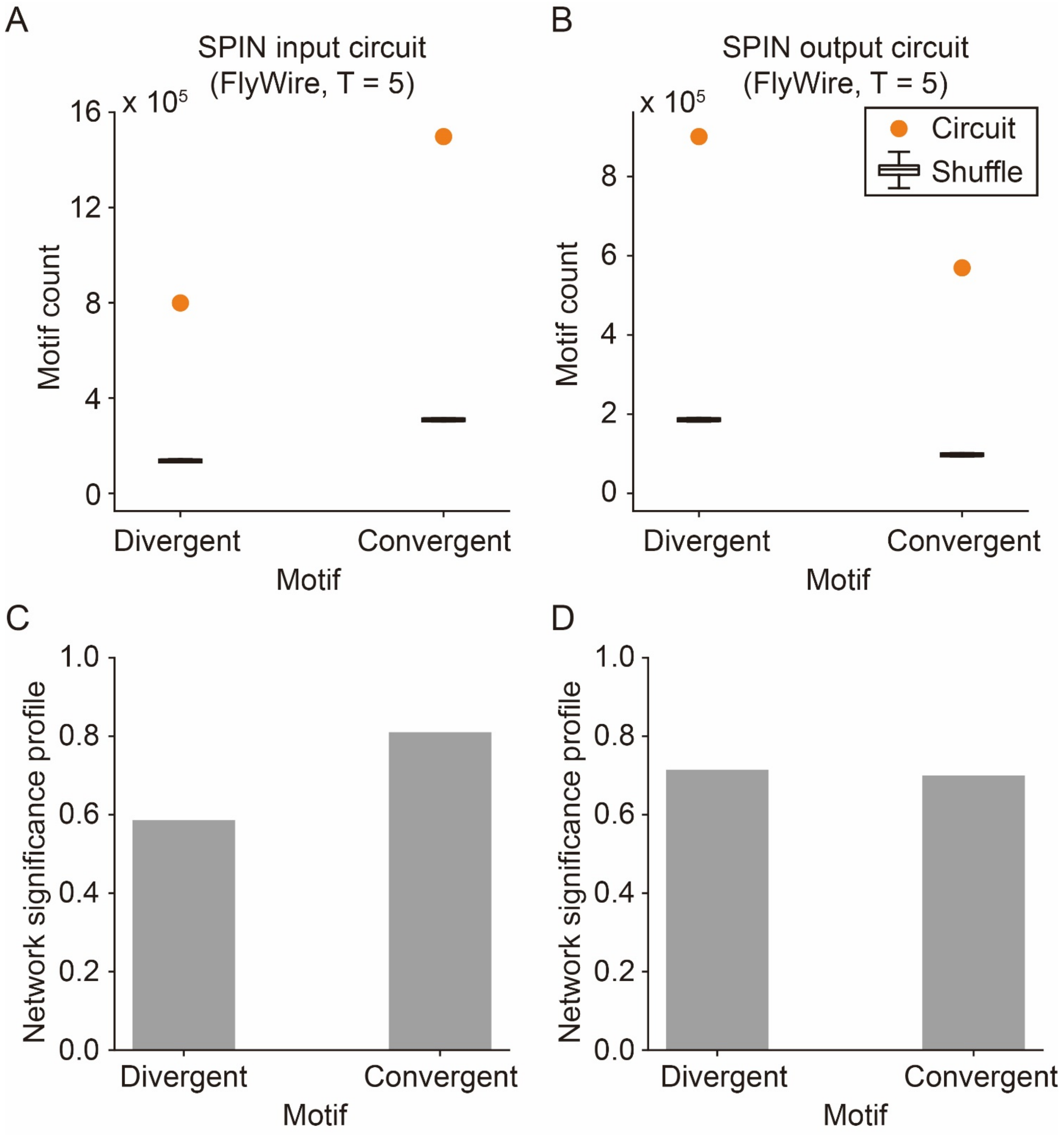
A
SPIN input circuit
(FlyWire, T = 5)
x 10^5
Motif count
Divergent
Convergent
Motif
B
SPIN output circuit
(FlyWire, T = 5)
Circuit
Shuffle
C
Network significance profile
D

Figure S4. Motif analysis of SPIN circuits in FlyWire at a synapse threshold of 5. (A-B) Divergence and convergence triplet motif counts in SPIN input **(A)** and output **(B)** circuits. Orange dots denote the counts of each triplet motif, while the box plots show the count distribution of 100 times of random reshuffling. **(C–D)** NSP of divergent and convergent motifs of SPIN input **(C)** and output **(D)** circuits in FlyWire at T = 5. The convergence-divergence pattern is consistent with the analysis at T = 2 **(Figure 2D, G)**. Although the pattern of network significance profile of output motifs is less prominent than at T = 2 due to the reduced number of retained connections, the overall qualitative pattern is preserved.

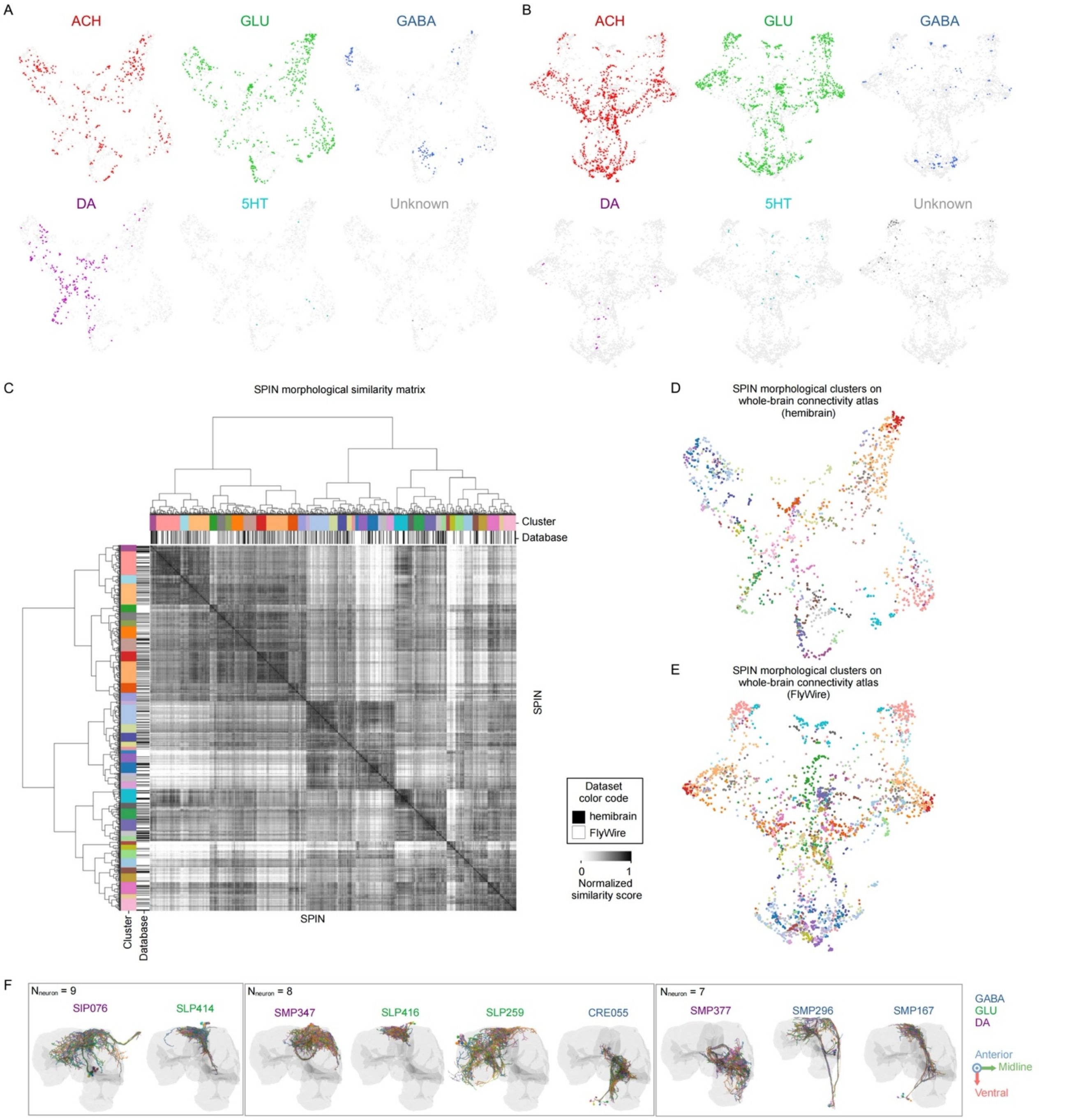
A
ACH
GLU
GABA
DA
5HT
Unknown
B
ACH
GLU
GABA
DA
5HT
Unknown
C
SPIN morphological similarity matrix
Cluster
Database
SPIN
SPIN
Cluster
Database
Dataset color code
hemibrain
FlyWire
0
1
Normalized similarity score
D
SPIN morphological clusters on whole-brain connectivity atlas (hemibrain)
E
SPIN morphological clusters on whole-brain connectivity atlas (FlyWire)
F
N neuron = 9
SIP076
SLP414
N neuron = 8
SMP347
SLP416
SLP259
CRE055
N neuron = 7
SMP377
SMP296
SMP167
GABA
GLU
DA
Anterior
Midline
Ventral

Figure S5. Neurotransmitter and cell type analysis of SPINs. (A-B) Whole-brain connectivity atlases highlighting SPINs of different neurotransmitters in hemibrain **(A)** and FlyWire **(B)**. Also, see Figure 2J (hemibrain) and 2K (FlyWire), which have the same color code. **(C)** Morphological similarity matrix of all SPINs jointly in both hemibrain and FlyWire datasets, color-coded by hierarchical clustering results. SPINs from both hemibrain and FlyWire datasets can be morphologically clustered together, indicating that many SPINs are traced in both datasets. **(D-E)** SPIN whole-brain connectivity atlas in hemibrain **(D)** and FlyWire **(E)**, color-coded by morphological clusters in **(C)**. **(F)** Morphology of SPIN types containing 9, 8, or 7 cells per type in anterior view in hemibrain. Cells are randomly colored. Cell-type legends are colored by their neurotransmitters. All these cell types are either dopaminergic or inhibitory.

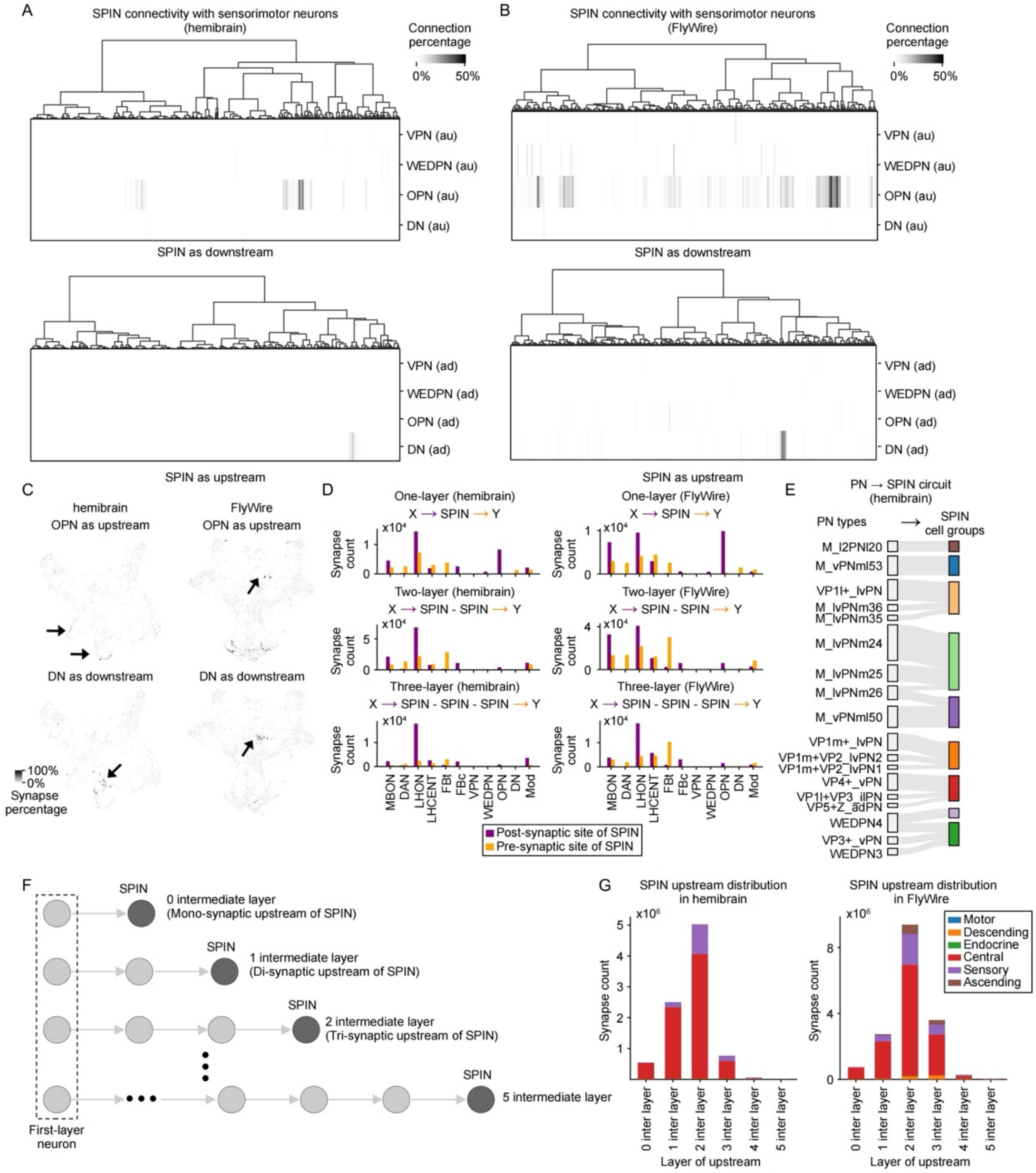
A
SPIN connectivity with sensorimotor neurons (hemibrain)
Connection percentage
0%
50%
VPN (au)
WEDPN (au)
OPN (au)
DN (au)
SPIN as downstream
VPN (ad)
WEDPN (ad)
OPN (ad)
DN (ad)
SPIN as upstream
B
SPIN connectivity with sensorimotor neurons (FlyWire)
C
hemibrain
OPN as upstream
FlyWire
OPN as upstream
DN as downstream
100%
0%
Synapse percentage
D
One-layer (hemibrain)
X → SPIN → Y
Two-layer (hemibrain)
X → SPIN - SPIN → Y
Three-layer (hemibrain)
X → SPIN - SPIN - SPIN → Y
One-layer (FlyWire)
Two-layer (FlyWire)
Three-layer (FlyWire)
Synapse count
MBON
DAN
LHON
LHCENT
FBt
FBc
VPN
WEDPN
OPN
DN
Mod
Post-synaptic site of SPIN
Pre-synaptic site of SPIN
E
PN → SPIN circuit (hemibrain)
PN types
SPIN cell groups
M_l2PNl20
M_vPNml53
VP1l+_lvPN
M_lvPNm36
M_lvPNm35
M_lvPNm24
M_lvPNm25
M_lvPNm26
M_vPNml50
VP1m+_lvPN
VP1m+VP2_lvPN2
VP1m+VP2_lvPN1
VP4+_vPN
VP1l+VP3_ilPN
VP5+Z_adPN
WEDPN4
VP3+_vPN
WEDPN3
F
SPIN
0 intermediate layer
(Mono-synaptic upstream of SPIN)
1 intermediate layer
(Di-synaptic upstream of SPIN)
2 intermediate layer
(Tri-synaptic upstream of SPIN)
5 intermediate layer
First-layer neuron
G
SPIN upstream distribution in hemibrain
SPIN upstream distribution in FlyWire
Synapse count
Motor
Descending
Endocrine
Central
Sensory
Ascending
0 inter layer
1 inter layer
2 inter layer
3 inter layer
4 inter layer
5 inter layer
Layer of upstream

Figure S6. SPIN connectivity with sensory and motor neurons. (A-B) SPIN connectivity strengths with major cell groups in the sensorimotor pathway as upstream (upper panel) or downstream (lower panel) in hemibrain **(A)** and FlyWire **(B)**. Each column is a SPIN. Also, see Figure 1G-J for those with MB, LH, FB, and modulatory neurons. **(C)** Sensorimotor-related SPIN connectivity strengths with OPN as upstream (upper) or DN as downstream (lower) in hemibrain (left) and FlyWire (right). **(D)** One- (upper row, X→SPIN→Y), two- (mid row, X→SPIN→SPIN→Y), and three- (lower row, X→SPIN→SPIN→SPIN→Y) layer connectivity preference of sensorimotor-related SPINs with major cell groups as upstream (magenta) or downstream (orange), based on synapse counts in hemibrain (left column) and FlyWire (right column). **(E)** One-to-one connectivity from mPNs and WEDPNs to sensorimotor-related SPINs in hemibrain. SPINs are clustered and color-coded by connectivity (see Methods). Connections with over 50 synapses are shown. **(F)** Schematic of SPIN multi-layer input circuits with 0-5 intermediate layers. **(G)** First-layer neuron superclass distribution of SPIN multi-layer input circuits in hemibrain (left panel) and FlyWire (right panel), color-coded by the superclass. Sensory neurons (purple) are particularly abundant in disynaptic and trisynaptic circuits in both datasets.

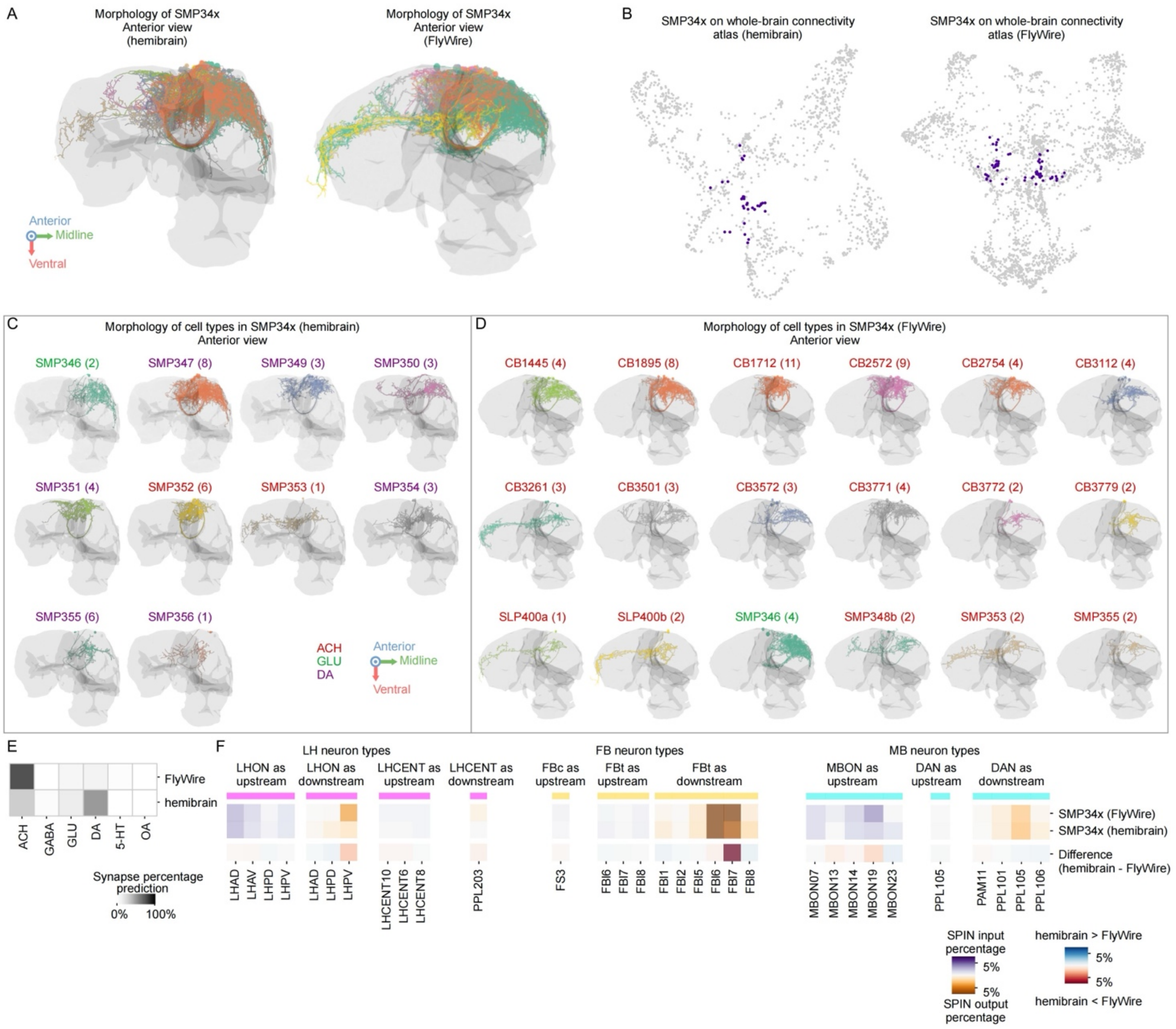
A
Morphology of SMP34x Anterior view (hemibrain)
Morphology of SMP34x Anterior view (FlyWire)
Anterior
Midline
Ventral
B
SMP34x on whole-brain connectivity atlas (hemibrain)
SMP34x on whole-brain connectivity atlas (FlyWire)
C
Morphology of cell types in SMP34x (hemibrain) Anterior view
SMP346 (2)
SMP347 (8)
SMP349 (3)
SMP350 (3)
SMP351 (4)
SMP352 (6)
SMP353 (1)
SMP354 (3)
SMP355 (6)
SMP356 (1)
ACH
GLU
DA
Anterior
Midline
Ventral
D
Morphology of cell types in SMP34x (FlyWire) Anterior view
CB1445 (4)
CB1895 (8)
CB1712 (11)
CB2572 (9)
CB2754 (4)
CB3112 (4)
CB3261 (3)
CB3501 (3)
CB3572 (3)
CB3771 (4)
CB3772 (2)
CB3779 (2)
SLP400a (1)
SLP400b (2)
SMP346 (4)
SMP348b (2)
SMP353 (2)
SMP355 (2)
E
FlyWire
hemibrain
ACH
GABA
GLU
DA
5-HT
OA
Synapse percentage prediction
0%
100%
F
LH neuron types
LHON as upstream
LHON as downstream
LHCENT as upstream
LHCENT as downstream
LHAD
LHAV
LHPD
LHPV
LHAD
LHPD
LHPV
LHCENT10
LHCENT6
LHCENT8
PPL203
FB neuron types
FBc as upstream
FBt as upstream
FBt as downstream
FS3
FBl6
FBl7
FBl8
FBl1
FBl2
FBl5
FBl6
FBl7
FBl8
MB neuron types
MBON as upstream
DAN as upstream
DAN as downstream
MBON07
MBON13
MBON14
MBON19
MBON23
PPL105
PAM11
PPL101
PPL105
PPL106
SMP34x (FlyWire)
SMP34x (hemibrain)
Difference (hemibrain - FlyWire)
SPIN input percentage
5%
5%
SPIN output percentage
hemibrain > FlyWire
5%
5%
hemibrain < FlyWire

Figure S7. Morphology and connectivity comparison of the SMP34x ensemble in hemibrain and FlyWire. (A) Anterior view morphology of SMP34x ensemble neurons in hemibrain (left) and FlyWire (right), colored by cell types, also see anterior view morphology for each cell type in panel C (hemibrain) and D (FlyWire). **(B)** SMP34x on whole-brain connectivity atlas in hemibrain (left) and FlyWire (right). **(C-D)** Anterior view morphology of all cell types in SMP34x ensemble in hemibrain **(C)** and FlyWire **(D)**, colored by types. Texts for cell types colored by neurotransmitters. The numbers of cells for each cell type are shown in brackets. **(E)** Comparison of SMP34x averaged predicted proportions of different transmitters between hemibrain and FlyWire. **(F)** Comparison of SMP34x connectivity with major cell groups between FlyWire and hemibrain. Each square denotes synaptic connection percentage averaged across all SMP34x neurons with each major cell type for both datasets, as well as their differences. We classified LHON neurons according to their arborizations in LH (LHAD, LHAV, LHPD, LHPV). We also classified FBt neurons according to their layers (FBl1-9).

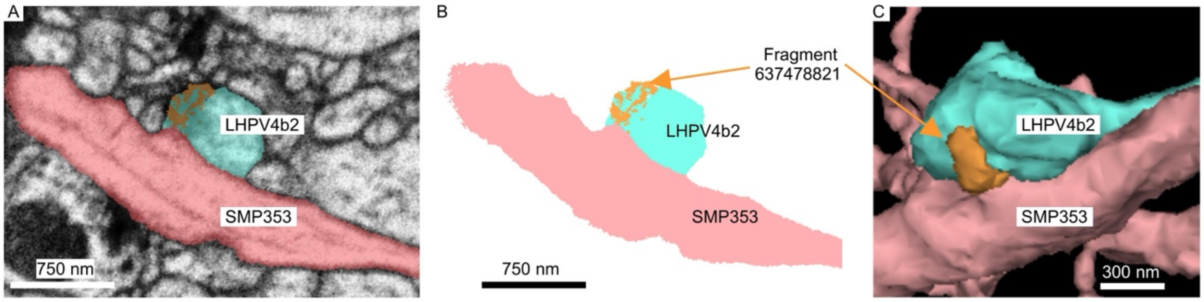
A
LHPV4b2
SMP353
750 nm
B
Fragment
637478821
LHPV4b2
SMP353
750 nm
C
LHPV4b2
SMP353
300 nm

Figure S8. Raw EM images of a single LHPV4b2 to SMP353 synapse from hemibrain. (A) EM image of a LHPV4b2 to SMP353 synapse with overlaying cell segmentation. LHPV4b2 and SMP353 are highlighted in pink and blue shades, respectively. Part of SMP353 postsynaptic density (yellow) is not well segmented. Scale bar: 300 nm. **(B)** Segmented fragments in **(A)**, including LHPV4b2 and SMP353, as well as a small fragment (yellow, 637478821) corresponding to the improperly segmented postsynaptic density. These segmentations were used for 3D reconstruction in Figure 5I-L. **(C)** 3D reconstruction of LHPV4b2, SMP353, and the small fragment. The small fragment due to improper segmentation causes the reconstructed SMP353 spine-like protrusion to be smaller than reality.

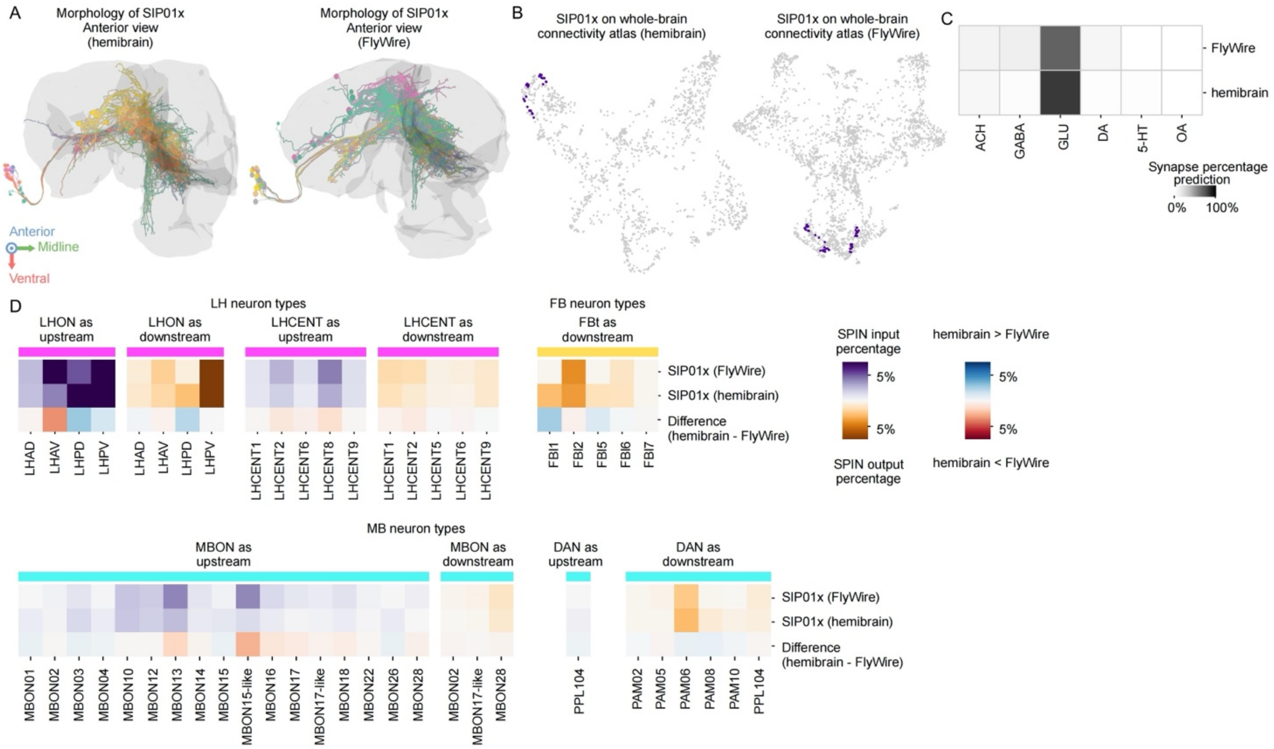
A
Morphology of SIP01x
Anterior view
(hemibrain)
Morphology of SIP01x
Anterior view
(FlyWire)
Anterior
Midline
Ventral
B
SIP01x on whole-brain
connectivity atlas (hemibrain)
SIP01x on whole-brain
connectivity atlas (FlyWire)
C
FlyWire
hemibrain
ACH
GABA
GLU
DA
5-HT
OA
Synapse percentage
prediction
0%
100%
D
LH neuron types
LHON as upstream
LHON as downstream
LHCENT as upstream
LHCENT as downstream
FB neuron types
FBt as downstream
SIP01x (FlyWire)
SIP01x (hemibrain)
Difference
(hemibrain - FlyWire)
LHAD
LHAV
LHPD
LHPV
LHCENT1
LHCENT2
LHCENT6
LHCENT8
LHCENT9
LHCENT5
FBl1
FBl2
FBl5
FBl6
FBl7
SPIN input percentage
5%
5%
SPIN output percentage
hemibrain > FlyWire
5%
5%
hemibrain < FlyWire
MB neuron types
MBON as upstream
MBON as downstream
DAN as upstream
DAN as downstream
MBON01
MBON02
MBON03
MBON04
MBON10
MBON12
MBON13
MBON14
MBON15
MBON15-like
MBON16
MBON17
MBON17-like
MBON18
MBON22
MBON26
MBON28
PPL104
PAM02
PAM05
PAM06
PAM08
PAM10

Figure S9. Morphology and connectivity of the SIP01x ensemble in hemibrain and FlyWire. (A) Anterior view morphology of SIP01x ensemble in hemibrain (left panel) and FlyWire (right panel), colored by cell type. **(B)** SIP01x on whole-brain connectivity atlas in hemibrain (left panel) and FlyWire (right panel). **(C)** Comparison of SIP01x averaged predicted proportions of different transmitters between hemibrain and FlyWire. **(D)** Comparison of SIP01x connectivity with major cell groups between FlyWire and hemibrain. Each square denotes the synaptic connection percentage averaged across all SIP01x neurons with each major cell type for both datasets, as well as their differences. We classified LHON neurons according to their arborizations in LH (LHAD, LHAV, LHPD, LHPV). We also classified FBt neurons according to their layers (FBl1-9).

2 Supplementary Tables

Brain regions	AL	antennal lobe
	AOTU	anterior optic tubercle
	ATL	antler
	AVLP	anterior ventrolateral protocerebrum
	CRE	crepine
	EB	ellipsoid body
	FB	fan-shaped body
	IB	inferior bridge
	ICL	inferior clamp
	LAL	lateral accessory lobe
	LH	lateral horn
	LO	lobula
	MB	mushroom body
	PLP	posteriorlateral protocerebrum
	PVLP	posterior ventrolateral protocerebrum
	SCL	superior clamp
	SIP	superior intermediate protocerebrum
	SLP	superior lateral protocerobrum
	SMP	superior medial protocerebrum
	SNP	superior neuropils
	SP	superior protocerebrum
	SPS	superior posterior slope
	VES	vest
	WED	wedge
Neurons	DAN	Dopaminergic neuron
	DN	descending neuron
	FBc	FB columnar neuron
	FBt	FB tangential neuron
	LHCENT	LH centrifugal neuron
	LHON	LH output neuron
	MBON	MB output neuron
	OPN	olfactory projection neuron
	uPN	uniglomerular olfactory projection neuron
	mPN	multiglomerular olfactory projection neuron
	SPIN	SP intrinsic neuron
	VPN	visual projection neuron
	WEDPN	wedge projection neuron

Supplementary Table 1. Abbreviations of brain regions and neuron types used in this manuscript.

Supplementary Videos

Supplementary Video 1. 3D view of SPIN whole-brain connectivity atlas via UMAP in hemibrain. Each dot denotes a single SPIN. SPINs are color-coded by 15 clusters based on brain-wide connectivity. See Figure 1K for the color code. See Methods for details.

Supplementary Video 2. 3D view of SPIN whole-brain connectivity atlas via UMAP in hemibrain. Each dot denotes a single SPIN. SPINs are color-coded by the SPIN connectivity preference of LH, MB, and FB. See Figure 1L for the color code.

Supplementary Video 3. 3D view of SPIN whole-brain connectivity atlas via UMAP in hemibrain. Each dot denotes a single SPIN. SPINs are color-coded by neurotransmitters. Also see Figure 2J.
